# The yellow perch (*Perca flavescens*) microbiome revealed resistance to colonisation mostly associated with neutralism driven by rare taxa under cadmium disturbance

**DOI:** 10.1186/s42523-020-00063-3

**Published:** 2021-01-05

**Authors:** Bachar Cheaib, Hamza Seghouani, Martin Llewellyn, Katherine Vandal-Lenghan, Pierre-Luc Mercier, Nicolas Derome

**Affiliations:** 1 Institut de Biologie Intégrative et des Systèmes (IBIS), Pavillon Charles-Eugène Marchand, Université Laval, 1030, avenue de la Médecine, Québec, QC G1V 0A6 Canada; 2 Institute of Biodiversity, Animal Health and Comparative Medicine (BACHM), Glasgow, University of Glasgow, Glasgow, UK; 3 School of Engineering, University of Glasgow, Glasgow, G12 8QQ UK

## Abstract

**Background:**

Disentangling the dynamics of microbial interactions within communities improves our comprehension of metacommunity assembly of microbiota during host development and under perturbations. To assess the impact of stochastic variation of neutral processes on microbiota structure and composition under disturbance, two types of microbial habitats, free-living (water), and host-associated (skin and gut) were experimentally exposed to either a constant or gradual selection regime exerted by two sublethal cadmium chloride dosages (CdCl_2_). Yellow Perch (*Perca flavescens*) was used as a piscivorous ecotoxicological model. Using 16S rDNA gene based metataxonomics, quantitative diversity metrics of water, skin and gut microbial communities were characterized along with development and across experimental conditions.

**Results:**

After 30 days, constant and gradual selection regimes drove a significant alpha diversity increase for both skin and gut microbiota. In the skin, pervasive negative correlations between taxa in both selection regimes in addition to the taxonomic convergence with the environmental bacterial community, suggest a loss of colonisation resistance resulting in the dysbiosis of yellow perch microbiota. Furthermore, the network connectivity in gut microbiome was exclusively maintained by rare (low abundance) OTUs, while most abundant OTUs were mainly composed of opportunistic invaders such as *Mycoplasma* and other genera related to fish pathogens such as *Flavobacterium*. Finally, the mathematical modelling of community assembly using both non-linear least squares models (NLS) based estimates of migration rates and normalized stochasticity ratios (NST) based beta-diversity distances suggested neutral processes drove by taxonomic drift in host and water communities for almost all treatments. The NLS models predicted higher demographic stochasticity in the cadmium-free host and water microbiomes, however, NST models suggested higher ecological stochasticity under perturbations.

**Conclusions:**

Neutral models agree that water and host-microbiota assembly promoted by rare taxa have evolved predominantly under neutral processes with potential involvement of deterministic forces sourced from host filtering and cadmium selection. The early signals of perturbations in the skin microbiome revealed antagonistic interactions by a preponderance of negative correlations in the co-abundance networks. Our findings enhance our understanding of community assembly host-associated and free-living under anthropogenic selective pressure.

## Background

Microorganisms drive the biogeochemical cycles of the earth and contribute towards homeostasis, immunity, physiology, behaviour [1, 2] and development [2, 3] across a wide range of metazoan hosts [4]. Host-microbiota symbioses involve complex and dynamic associations between obligate and facultative symbionts [5]. Disentangling the dynamics of microbial interactions within communities improves our comprehension of metacommunity assembly [6]. Ecological processes (i.e., dispersal, selection and ecological drift) shape these interactions and govern the assembly rules of the ecological communities [7, 8]. The impact of ecological processes on community assembly is a long-term debate in macroecology. Stochastic processes related to the neutral theory of biodiversity suggests no impact of ecological interactions on species distribution and abundances. Neutrality reflects that diversity units “Species” are ecologically equivalent, while stochasticity implies random variation in mean demographic rates [9]. In such cases, local communities are randomly connected to a single metacommunity through differing rates of migration, death and birth [10–12]. In contrast, deterministic related niche theory considers that environmental conditions and interspecific interactions, including competitive exclusion, determine distribution and abundance of species [13]. The niche processes imply differentiation in mean demographic rates, while ‘determinism’ reflects an absence of random variation in species’ demographic rates. In microbial ecology, the advent of culture-independent approaches such as high-throughput 16S rDNA metabarcoding paved the way for the conceptual framework of the Operational Taxonomic Unit (OTU) or Amplicons Sequences Variants (ASVs) used as units of microbial diversity. Such advancements have opened new mathematical [14–19] and network-based [6, 20, 21] models for predicting ecological interactions between microbial communities. These models helped in constructing hypotheses on types of processes driving microbiomes assemblies over evolutionary time.

Models for quantifying the neutral [14, 16, 22, 23] and deterministic [17, 24–26] processes in different types of microbial ecosystems continue to provide new comprehensive insights regarding the forces governing microbiome assembly. Both neutral and non-neutral processes have been evidenced as drivers of the microbial metacommunity assembly in many vertebrate microbiomes [27, 28], as well as in the natural environment [29]. For instance, neutral processes were identified as playing a major role during the development of host-associated microbial communities in different domesticated vertebrate and plant models [28, 30, 31]. When focusing on case-control surveys, the influence of selective pressure on microbiota assembly is more salient. In contrasting contexts such as human oral and gut microbiome under antibiotic therapy [32] and euryhaline fish microbiome during salinity acclimation [33], the community assembly was potentially driven by deterministic processes, with little evidence for stochastic colonisation. Nonetheless, there is much work to do to fairly understand the microbial assembly under disruptive conditions across a diverse range of host species. In the present study, we first predict that a selection gradient induced by a concentration gradient of a toxic metal will not only disrupt the host physiology, [34] but will also overwhelm the assembly of symbiont consortia. Second, we predict that the host will lose its filtering capacity to recruit the appropriate symbionts, which in turn, will translate into increased colonisation of opportunistic strains, as predicted by the colonisation resistance model [32]. Third, from the interaction networks of OTUs, we expected that the low abundant taxa might play an important role in the metacommunity assembly [35–39]. To this end, we measured the effect of directional selection along the developmental stages of the host organism -Yellow Perch juveniles (*Perca flavescens*) were exposed to two selection regimes: a constant (9 ppb) and a gradual (0.8 to 9 ppb) exposure to non-lethal doses of cadmium chloride (CdCl_2_), over 90 days. The taxonomic compositional dynamic of two types of microbial habitats, free-living (water), and host-associated (skin and gut), were characterised throughout the young developmental stages of the host using a 16S SSU rDNA gene, based metataxonomics approach [40]. Being able to cope with polymetallic gradients generated by acid mine drainages (AMD), the Yellow Perch is a well-established ecotoxicology vertebrate model: many studies have measured the impact of heavy metals on their innate immune system [41], metabolism [42], development [43], parasitism [44–46] and gene expression [47]. As these host functions are closely related to gut microbiota composition, the Yellow Perch is a convenient model to test the effects of metal exposure on a vertebrate host-microbiota system. Impact of Cd exposure on microbial communities was documented for AMD bacterioplankton [48], but not within the host-microbiota system. Using non-lethal doses of Cd as a selection pressure, we sought to unravel the neutral and non-neutral processes shaping the microbiota assembly, without triggering significant physiological damage or causing host death.

## Materials and methods

### Fish rearing

After an acclimation period of 30 days in two containers of 1500 L each, the Yellow Perch (1200 juveniles) were reared in 24 tanks (50 fish per tank) of 36 L and acclimated for the second period of 15 days. Each tank was equipped with an independent filtering system circuit. The fish juveniles were fed daily with the same food from the beginning to the end of the experiment. A second acclimation period of 2 weeks was carried out before the start of Cd exposure (Supplementary file 1).

### Cadmium exposure regimes

Control (Control) and Cd (cadmium) treated tanks were randomly distributed in the aquarium facility. The experiment was designed for two Cd exposure regimes (8 tanks per regime), and one negative control regime (8 tanks). In treated tanks, fish were exposed to Cd chloride (CdCl_2_) provided by Sigma-Aldrich (> 99.9% purity). The Cd was dissolved in ddH2O to target stock concentrations (9 ppb). For the regime of Cd constant concentration (CC), the Cd chloride was initially added at 0.8 ppb (parts per billion), before gradually increasing the concentration every 5 days to reach a maximal concentration at the end of the first month (T1). This maximal concentration was maintained 2 months until the end of treatment (third month, T3). For the regime of Cd variable concentration (CV), the Cd concentration was gradually increased every 5 days to reach the target concentration (9 ppb) at the end of treatment (third month, T3). The maximal CdCl_2_ concentration was set at 9 μg/L as it is the highest CdCl_2_ concentration tolerated by Yellow Perch in contaminated Canadian lakes [49].

### Host-microbiota and water sampling

A total of 432 mucosa host-microbiota samples were collected for this study, 216 (3 times × 3 regimes × 8 tanks × 3 replicates) skin mucus swabs and 216 (3 times × 3 regimes × 8 tanks × 3 replicates) gut tract samples (Supplementary file 2). Water samples were stored in sterile bottles (Nalgene), 2 l per tank were filtered using a polycarbonate membrane of 0.22 μm. In total, 144 filters (3 times × 3 regimes × 8 tanks × 2 replicates) were conserved in 2 mL sterile microcentrifuge tube and directly stored at − 80 C.

### Metal concentration in water and fish liver

Concentrations of metal traces (Cd, Cu and Zn) within the water and liver were determined with the ICPMS (Ionization Coupled Mass spectrometry) technology at the Department of Chemistry, Laval University for T0 and T1, then at INRS (*Institut National de la Recherche Scientifique*), Quebec, for T1-T3. For water, before ICPMS analysis of Cd ions, the CdCl_2_ in water samples (10 ML tubes) was fixed by adding 4% of nitric acid. The metals ions (Cd, Cu, Zn) concentrations were measured in water every week until the end of the CdCl_2_ exposure regimes. For fish, liver samples after lyophilisation were digested with purified nitric acid and kept at room temperature for 5 days. The liver acid digestion protocol was adapted from Pierron et al. [50]. For further details, see the Supplementary file 2. The metal concentrations were analysed using a two-way analysis of variance (ANOVA) of two independent factors: time and treatment. The interactions between time and treatment factors (the interaction means that the effect of treatment depends on time) were analysed using Tukey’s test and Wilcoxon rank test depending on the data (metal concentration) after assessing the normality of data distribution.

### DNA extraction to Illumina Miseq sequencing

DNA was extracted from all skin mucus and water samples using the Qiagen DNeasy Blood and tissue kit (Supplementary file 2). For all intestine samples, after an RNA extraction for a transcriptomic project, the DNA was extracted from TRIzol organic phase using BEB (back extraction buffer) and PCI (phenol/chloroform/isoamyl alcohol 25:24:1) solution (Supplementary file 3). The 16S ribosomal DNA was amplified via PCR using universal primers specific to the V3-V4 hypervariable region of the rDNA 16S gene [51]. The purified product of first-round PCR was used as a template for the library preparation by performing second-round PCR. Final amplified DNA was verified by electrophoresis on 2% agarose gel, and finally, DNA concentration of the product was quantified by fluorescence using Quant-iT™ PicoGreen™ dsDNA Assay Kit (Thermo Fischer Scientific).

### Analysis of 16S rDNA amplicons

Sequence analysis was performed with our bioinformatic pipeline as described previously [27, 40]. The code of our pipeline is available on Github (https://github.com/BachBioinformatics/MicrobiomePipelines). After the construction of OTUs table, a decontamination step was performed using BWA mapper [52] implemented in DeConseq tool [53], which consisted of mapping the OTUs sequences against the draft genome of *Perca flavescens* available on NCBI (98% identity threshold). The analysis of the alpha and beta diversity of metacommunities was proceeded using the Rhea package [54]. Afterwards, the significance of variations in alpha-diversity indexes (richness and evenness) and beta-diversity (phylogenetic distance) divergence between experimental groups was assessed using pairwise and multiple rank statistics tests (Wilcoxon /Kruskal-Wallis). Beta-diversity was measured using generalised UniFrac distance [55], which considers both dominant and rare OTUs. *P*-values of pairwise comparisons in alpha and beta-diversity were validated with multiple correction tests [56] using the B-H (Benjamini-Hochberg) for avoiding the Type I errors (false positives).

### Correlational networks analyses

The Spearman coefficients supported with multiple corrections test Benjamini-Hochberg (BH) were computed to measure the correlations between OTUs. This coefficient was recently demonstrated as a robust approach in terms of sensitivity and precision of correlation detection [57]. Only strong correlations (*p*-value BH < 0.05) positive (Corr > 0.6), and negative (Corr < − 0.6) were visualized in the OTU networks. Cytoscape software [58] was used to perform network visualization and analysis. The number of components indicated the fragmentation (number of subnetworks) in each community per condition. Each node size in the network was proportional to the average OTU’ relative abundance in all the samples per group. Although Spearman and new methods of networks inference [57] are comparable, possible bias of indirect associations of nodes can be generated by spearman coefficient [59]. To deal with this bias, the SPIEC-EASI (SParse Inverse Covariance Estimation for Ecological Association Inference) method [59] was used to supplement the OTUs networks built with spearman coefficient. In SPIEC-EASI networks, to avoid false-negative correlations, the OTUs which occur in less than three samples were combined in one synthetic OTUs. To compare the connectivity of networks between different groups, the average of nodes degree was compared using the Kruskal-Wallis test. Also, the modularity of different networks was assessed with the Markov Clustering Algorithm (MCL) using a value of 2.5 as a reasonable inflation parameter (granularity) of clustering [60]. Modularity is a measure of network structure that was designed to measure the strength of division of a network into modules. Networks with high modularity have dense connections between the nodes within modules but sparse connections between nodes in different modules [61].

### Metacommunity assembly modelling

To investigate the role of neutral processes in community assembly, we fit the distribution of OTUs to a neutral model of microbial assembly [16] using a non-linear least-squares approach and beta distributions, which has recently been implemented by Burns and colleagues [22]. The neutral model compares the frequency of OTU occurrence to their abundance in the metacommunity by estimating a parameter (m), which represents the migration rate and which can be interpreted as a measure of dispersal limitation (low migration rate means high dispersal limitation) [22]. The estimated migration rate (m) is the probability that a random loss (death or emigration) of an OTU in a local community is replaced by dispersal from the metacommunity source [22]. The temporal comparisons (T0-T1; T1-T3) of predicted versus observed OTU frequencies from the neutral model were used to highlight the percentage of OTUs fitting the model with a confidence interval of 95%. The goodness of fit to the neutral model was assessed using R-square as the coefficient of determination. R-squared equal or higher to a value = 0.5 is an acceptable threshold of the goodness of fit to Sloan’s neutral model [16]. In addition to Sloan’s model, to quantify the ecological stochasticity in the community structure, the new proposed index, normalized stochasticity ratio (NST) was computed to estimate the ecological stochasticity based on beta diversity [62]. The NST index was used here to estimate the average stochasticity between local communities (treatments and control) using Jaccard and Ruzicka metrics (recommended by the authors of NST model) and “fixed proportion” as null-model which means that the occurrence probability of a taxon is proportional to its observed occurrence frequency. The NST values were computed using the function “tNST”, then because pairwise comparisons of observed/null dissimilarity values are not independent, bootstrapping analysis based random comparisons (*N* = 1000) for stochasticity ratio was also performed.

## Results

### Metal concentrations, diversity measures and taxonomic composition

**Fig. 1:**
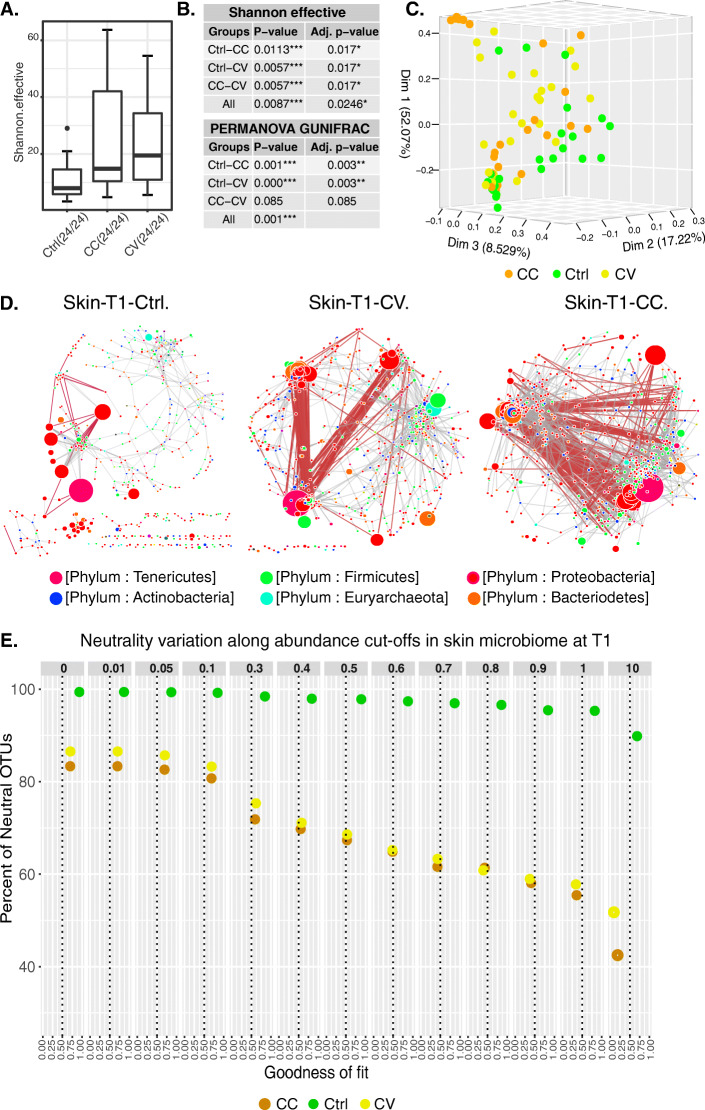
Diversity, structure, and assembly of the skin microbiome. **a** highlights the negative correlations in the co-abundance networks of skin microbial community. Each node size in the network is proportional to the average of the OTUs relative abundance in all samples. These networks are based on significant Spearman coefficients and were constructed using R scripts and Cytoscape software. **b** shows the boxplots of the significant difference in alpha-diversity (Shannon effective). **c** summarises the statistical tests of alpha-diversity and beta-diversity (Gunifrac distance). **d** represents the PCOA 3D plot of the microbial skin communities of all treatment groups based on the significant difference of generalized Unifrac distances tested with PERMANOVA, MRPP and multiple correction test (See Supplementary Table 3) **e** reports the distribution of neutrality versus abundance cut-off and goodness of fit. The plot shows the variation of neutral OTUs percentage (Y-axis) along with the goodness of fit predicted by NLS models using 12 cut-offs of relative abundance averages (facet panels) in the skin metacommunity at T1 and T3

**Fig. 2:**
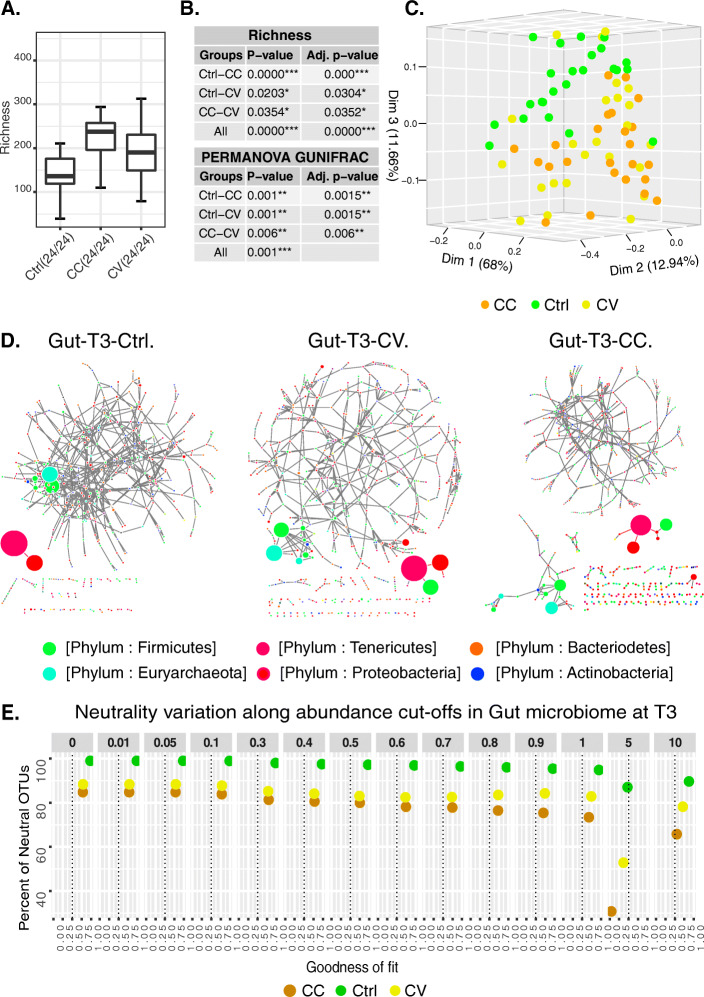
Diversity, structure, and assembly of the gut microbiome. **a** highlights the peripheral location of the most abundant OTUs in the correlation networks of the gut microbial community. Each node size in the network is proportional to the average of the OTUs relative abundance in all samples. These networks are based on significant Spearman coefficients and were constructed using R scripts and Cytoscape software. **b** shows the boxplots of the significant difference in alpha-diversity (Shannon effective). **c** summarizes the statistical tests of alpha-diversity and beta-diversity (Gunifrac distance). **d** represents the PCOA 3D plot of the microbial skin communities of all treatment groups based on the significant difference of generalized Unifrac distances tested with PERMANOVA, MRPP and multiple correction test (See Materials and Methods and Supplementary Table 3) (**e**) reports the distribution of neutrality versus abundance cut-off and goodness of fit. The plot shows the variation of neutral OTUs percentage (Y-axis) along with the goodness of fit predicted by NLS models using 12 cut-offs of relative abundance percentages (facet panels) in the gut metacommunity at T1 and T3

**Fig. 3:**
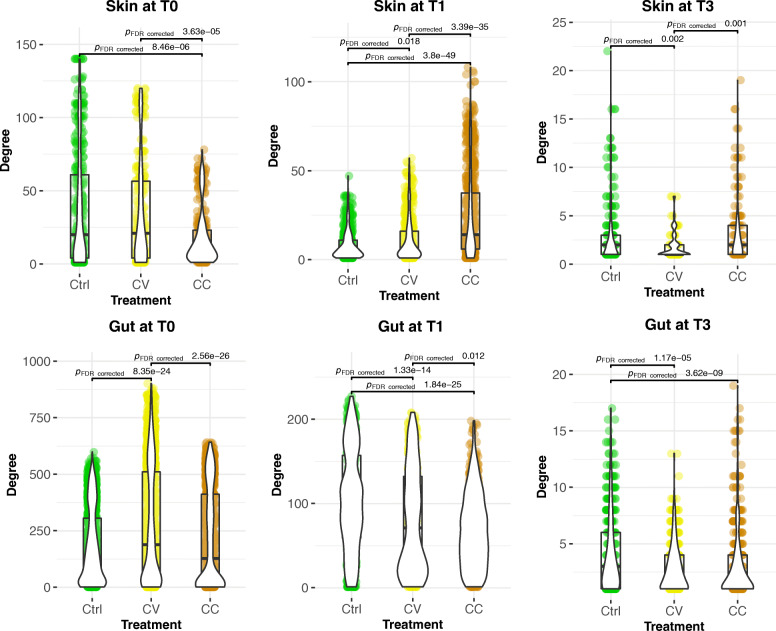
The average degree of host-microbiome networks over time and between treatments. The connectivity represented with violin plots are significantly higher in average for the control groups in the gut and the skin at time T3, whilst at T1, they were significantly higher for cadmium-treated groups only in the skin. The average of nodes’ degree computed with Network Analyzer was compared using the Kruskal-Wallis test followed by Benjamini-Hochberg test. The value of 0.05 is the threshold of B-H *p*-value significance. Only the significant Dunn test *p*-values for pairwise comparisons are displayed on this figure, however in order to improve visibility, the significant *p*-values of Kruskal-wallis test for multiple groups comparisons were not plotted

In water, the difference in Cd concentration is significant between all treatments (CC, CV or Control) at T1 and T3. In the fish liver, the difference is only significant at T3 (Supplementary Table 1**)**. For alpha-diversity, the treatment reveals a significant effect only on the evenness (*Shannon effective*) in the skin mucosal communities at T1, and on the richness in the gut communities at T3 (Figs. 1a-b and 2a-b). The Cd treatment reveals also a significant imbalance in the taxonomic composition in the skin microbiome at T1 (Table 1). The statistical comparisons using both diversity indices for evenness and richness demonstrate the importance of time as a driver in microbial community richness, evenness (Fig. 1a**,** Supplemetal Figure 1, Supplemetal Table 2**)**, and taxonomic composition (Supplemetal Figures 2 and 3**)** rather than treatment. For beta-diversity (GUniFrac distance), by T1, significant differences (BH *p-value* < 0.05 of significant PERMANOVA tests) among treatments are only observed in the mucosal skin communities (Supplemetal Table 3**,** Fig. 1b-c); however, by T3, both cadmium exposure regimes indicate significant changes in both skin and gut microbial communities compared to the control (Supplementary Table 3, Fig. 2b-c). Among water microbial communities, the phylogenetic distance between treatments and control is significantly different at every time points (BH’s *p-value* of PERMANOVA tests for CC-Ctrl _T1_: 0.0135; CC-Ctrl _T3_: 0.002; CC-CV _T1_: 0.136; CC-CV _T3_: 0.0015_;_ CV-Ctrl _T1_: 0.006; CV-Ctrl _T3_:0.0015). Interestingly, the phylogenetic distance between CV and CC treatments becomes significant at T3 (Supplementary Table 3**;** Supplementary Figure 4).

**Table 1:** Statistical summary of taxonomic significant changes between treatments

Phylum-Genus	Group1	Group2	Wilcoxon Rank Sum Test − pairwise *p*-value	Adj. *p* − value
p__Bacteroidetes	Ctrl	CC	0.0003 **	0.0004 **
	Ctrl	CV	0.0001 ***	0.0001 ***
	CC	CV	0.767	0.767
g__Emticicia	Ctrl	CC	0.0005 **	0.0008 **
	Ctrl	CV	0.0001 ***	0.0003 **
	CC	CV	0.891	0.891
g__Flavobacterium	Ctrl	CC	0.0028 *	0.0042 *
	Ctrl	CV	0.0001 ***	0.0003 **
	CC	CV	0.3305	0.3305
g__Pseudorhodobacter	Ctrl	CC	0.0065 *	0.0176 *
	Ctrl	CV	0.0117 *	0.0176 *
	CC	CV	0.5336	0.5336
g__Shinella	Ctrl	CC	0.0001 ***	0.0002 **
	Ctrl	CV	0.0001 ***	0.0002 **
	CC	CV	0.9268	0.9268
g__Sphaerotilus	Ctrl	CC	0.0039 *	0.0058 *
	Ctrl	CV	0.0006 **	0.0018 *
	CC	CV	0.736	0.736

This table resumes the main important significant changes of the taxonomic composition, at the phylum and genus levels, between treatments in the skin microbiome at time T1. Pairwise comparisons were performed using the Wilcoxon Rank Sum Test – pairwise. *P*-value < = 0.05: “*”;  *p-value* < = 0.001: “**” ; *p*-value < = 0.0001: “***”

Overall, the effect of treatment on the host-microbiota for beta diversity is more significant than time (i.e. microbiota ontogeny) for skin (T1, T3) and gut (T3) microbiota. Contrastingly, for alpha diversity, the effect of time is more significant than treatment. To explore the effect of Cd treatment on the interactions within and between host and water microbial communities we analyzed the networks parameters mainly degree and modularity.

### Substantial role of rare taxa in microbiome network connectivity

Within gut microbial communities, the high abundant OTUs are peripheral (with minimal interactions) or disassortative (not connected) to the giant network component, see [63], suggesting low overall connectivity (Fig. 2d**).** Within skin and gut microbiome networks, no correlation was found between the degree (number of connections per node) and the average relative abundance of OTUs (nodes size in the network). However, given that the high abundance is a feature of few OTUs, most of the connections within host-microbiome networks occur among rare or low abundant OTUs (relative abundance < 0.1%) – especially in skin communities early in both Cd exposure regimes a T1 in the networks of Cd treatment groups (Fig. 1d**).**

### Poor connectivity in gut microbiome network reflects cd selection regimes

The exposure to Cd has also a significant impact on network connectivity and integrity in the gut microbial communities. In the control group, most abundant OTUs were connected to a central hub (Fig. 2d). However, in the Cd-treated groups, the most abundant OTUs (> 1%) were gradually disconnected from the main network in small independent hubs or sub-networks. Considering the MCL clustering of gut microbiome networks into modules, the modularity indicates higher values at time T1 in Cd treatment groups (Ctrl _modules_ = 12; CV _modules_ = 41; CC _modules_ = 44). However, at time T3 the network’s modularity is higher in the control group (Ctrl _modules_ = 94; CV _modules_ = 50; CC _modules_ = 65) (Table 2; Supplementary Table 4). In term of nodes connectivity, the average degree was higher in the control group compared to Cd treatment groups at T1 and T3 (Fig. 3; Supplementary Table 4).

**Table 2:** **Host microbiome networks modularity**

Skin microbiome network	T0	T1	T3
Ctrl	78	47	42
CV	51	104	28
CC	56	149	30
**Gut microbiome network**	**T0**	**T1**	**T3**
Ctrl	375	12	94
CV	315	41	50
CC	445	44	65

This table resumes the number of modules obtained from MCL clustering of edge betweenness (see Materials and Methods) of skin and gut microbiome networks

### Negative correlations in the skin microbiome network suggest dysbiosis under disturbance

In skin microbial community, the correlational networks at time T1 are characterised by high modularity (Ctrl _modules_ = 12; CV _modules_ = 41; CC _modules_ = 42) (Table 1; Supplementary Table 4) and a significantly high average degree in the Cd treatment groups (Fig. 3). However, at time T3, like in the gut microbiome, the skin microbiome networks indicate higher modularity in the control group (Ctrl _modules_ = 42; CV _modules_ = 28; CC _modules_ = 30), and significantly high average degree. The higher average degree connectivity observed in CV and CC relative to the Control treatment at T1 is manifest in the significantly high percentage of significant negative correlations (red edges in networks of Fig. 1d) observed in those groups (CV _neg. corr._: 6.9%; CC _neg. corr._: 6.3%) compared to the control group (Control _neg. corr._: 2.2%). A significant increase over time (T0-T1) in the abundance of Tenericutes (T), and Bacteroidetes (B), and decrease of Firmicutes (F),and Proteobacteria (F) (Supplementary Table 5) is associated with nodes implicated with negative correlations in the control group Ctrl (T:6% B: 3% F: 6% P: 63%), CV (T:6% B:7% F: 19% P: 57%) and CC (T:8% B: % 7 F: 13% P: 61%). In fact, at the time T1, the Bacteroidetes have significantly increased over time in the skin for CC & CV groups (Supplementary Table 5), not for control, showing a significantly higher abundance in skin compared to water and gut microbial communities for Cd groups (Supplementary Figure 5). Like Bacteroidetes, few nodes are exclusively implicated in negative correlations in CC and CV for Synergistetes (~ 1%), Acidobacteria (~ 1%), and Deinococcus-Thermus (~ 1%). Interestingly, however, Euryarchaeota (E) is more implicated in negative correlations within the control group Ctrl (10%), than CV (2%) and CC (1%).

### Fragmentation of water microbiome network explains community dynamics

**Fig. 4:**
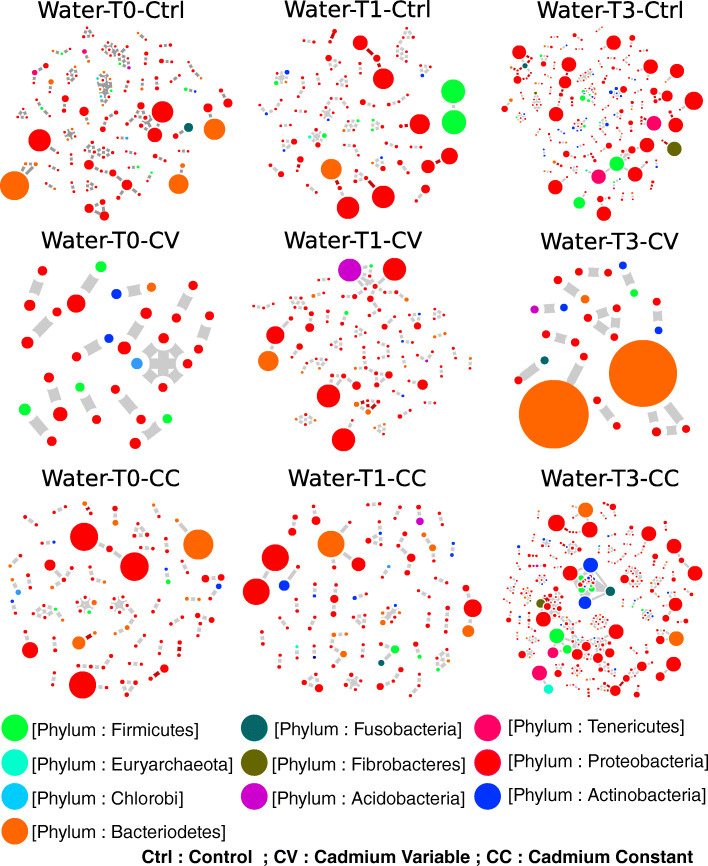
Correlational co-abundance networks of water microbial community. Water microbial community networks displayed fragmented interactions, over time and between treatments — the topology of water microbial encompassed sub-networks disconnected in small independent hubs. The number of independent hubs was the highest in Control network at T0 and T3, at T3 in CC, and it was always intermediate in CV network (see the Supplementary Table 3). These networks are based on significant Spearman coefficients and were constructed using R scripts and Cytoscape software

**Fig. 5:**
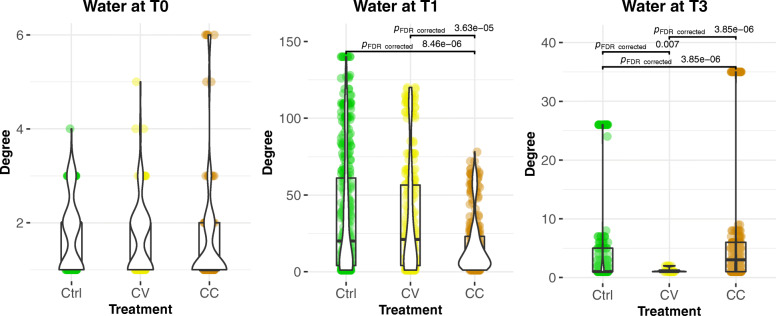
The average degree of water microbial networks over time and between treatments. The connectivity represented with violin plots is significantly higher on average for the control groups in the gut and the skin at times T3 and T1. The average of nodes’ degree computed with Network Analyzer was compared using the Kruskal-Wallis test followed by Benjamini-Hochberg test. The value of 0.05 is the threshold of B-H *p*-value significance. Only the significant Dunn test *p*-values for pairwise comparisons are displayed on this figure, however in order to improve visibility, the significant *p*-values of Kruskal-wallis test for multiple groups comparisons were not plotted

The water microbiome networks are fragmented, both over time and among treatments (Fig. 4; Supplementary Table 4). There was a notable lack of Tenericutes (*Mycoplasma*) in comparison to the skin and intestinal microbial communities’ networks. The degree average is significantly high in the control group of water microbiome networks at T1 and T3 (Fig. 5). Overall, the structure of the water networks encompassed small, disconnected worlds of independent hubs.

### Metacommunity dynamic indicates niche specialisation and time effect

**Fig. 6:**
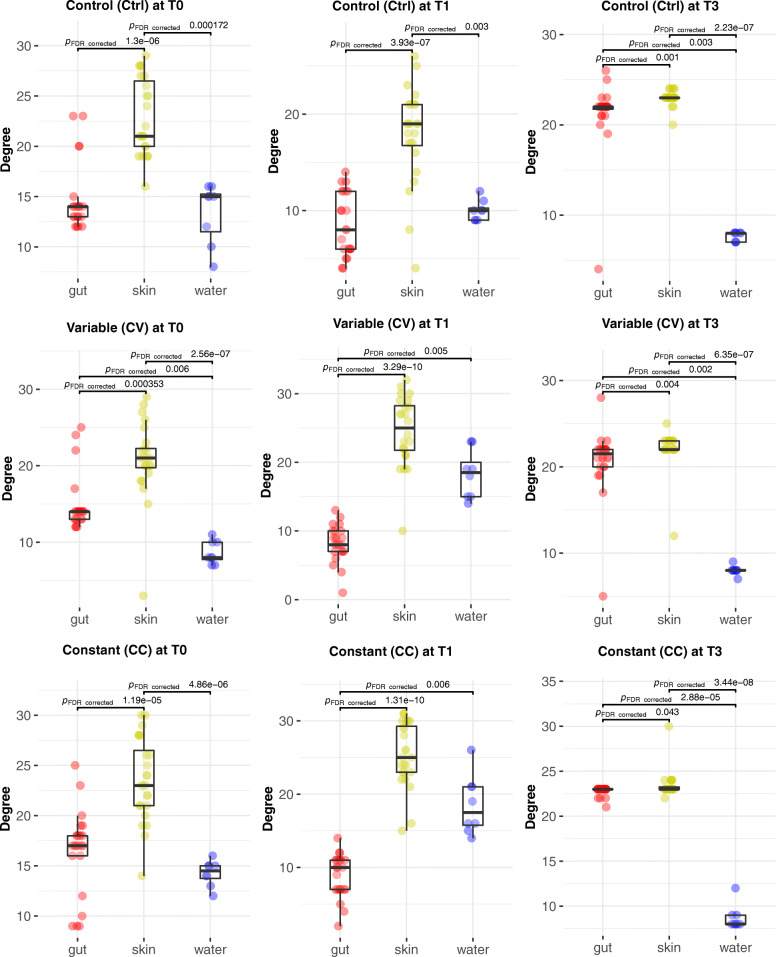
Statistical analysis of the node degree in the water and host-microbiome networks. The comparison of connectivity assessed with nodes degrees and represented with violin plots indicate that the average of connections is significantly higher in the skin compared to the water and gut microbiomes in all treatments and at all time points. However, at T1 the connectivity converged (which means not significantly different) between the water and skin microbiome for cadmium-treated groups. The average of nodes’ degree computed with Network Analyzer was compared using the Kruskal-Wallis test followed by Benjamini-Hochberg test. The value of 0.05 is the threshold of B-H *p*-value significance. Only the significant Dunn test *p*-values for pairwise comparisons are displayed on this figure, however in order to improve visibility, the significant *p*-values of Kruskal-wallis test for multiple groups comparisons were not plotted

The dynamics of interactions of water microbial communities with the different host (skin and gut) microbiomes reveal the similar structure of networks between treatments, but not overtime. At T1, the subnetwork assembling gut nodes is weakly connected to the modules of the water and skin microbial subnetworks. A T3, the three microbial subnetworks of water, gut and skin are almost disconnected within the Cd treatment groups and weakly connected in the control group (Fig. 6). The comparison of connectivity assessed with nodes degrees and represented with violin plots (Supplementary Figure 6**)** indicate that the average of connections is significantly higher in the skin compared to the water and gut microbiomes in all treatments and at all time points. However, at T1 the connectivity converged (which means not significantly different) between the water and skin microbiome for cadmium-treated groups. The comparison of centrality assessed with nodes closeness centrality and represented with violin plots (Supplementary Figure 7) indicates that the average of connections is significantly higher in the skin compared to the water and gut microbiomes in all treatments at T0 before disturbance. However, at T1 the average node centrality converged (which means not significantly different) between the water and skin microbiome for cadmium-treated groups. At T3, the centrality of node converged between the gut and skin microbiome in the control group. Finally, from a methodology point of view, the networks built with SPIEC-EASI method and Spearman coefficient converge to similar topologies (see the gut and skin microbiome networks built with SPEIC method in Supplementary Figure 8).

### Stochasticity and determinism involved in water and host-associated bacterial community assembly

In host and water bacterial communities, considering all the OTUs without any filtration of abundance, the occurrence frequency of the majority of OTUs in the Gut (CC_T1:T3_ = 93:85%; CV_T1:T3_ = 94:88%; Ctrl_T1:T3_ = 99.6:99.4%), the skin (CC_T1:T3_ = 83:91%; CV_T1:T3_ = 86:88%; Ctrl_T1:T3_ = 99.4:99.3%) and the water (CC_T1:T3_ = 81:99%; CV_T1:T3_ = 75:88%; Ctrl _T1:T3_ = 97:93%) was confidently predicted by the NLS model (Supplementary Table 6**,** Figs. 1e and 2e). According to this model, the percentage of neutral OTUs and the goodness of fit (R^2^) are always higher in the control group compared to both Cd-treated regimes in water and host communities (Supplementary Table 6). The percentage comparison of neutral OTUs between different models could not quantify the relative impact of the non-neutral OTUs (overfit or underfit the model) because the goodness of fit and the estimated emigration rate with maximum likelihood (m.mle) varied between water and host models (Supplementary Table 6). The emigration rate in the NLS model predicts the stochastic demography in community assembly. So, at T1, the comparison of the estimated emigration rates indicates that stochasticity was higher in the water microbiome compared to host microbial communities in the Control (Gut_T1_ = 0.172; Skin_T1_ = 0.200; Water_T1_ = 0.326), CV (Gut_T1_ = 0.118; Skin_T1_ = 0.204; Water_T1_ = 0.423) and CC (Gut_T1_ = 0.129; Skin_T1_ = 0.242; Water_T1_ = 0.335). Similarly, at T3, the migration rate indicates higher stochasticity in water compared to the host-microbial communities for the Control (Gut_T3_ = 0.269; Skin_T3_ = 0.177; Water_T3_ = 0.488), and CV (Gut_T3_ = 0.351; Skin_T3_ = 0.138; Water_T3_ = 0.424) groups, but not for the CC (Gut_T3_ = 0.564; Skin_T3_ = 0.169; Water_T3_ = 0.362). Investigating the relationship of OTU abundance and neutrality, our analysis indicates that the percentage of neutral OTUs decreased in the Ctrl groups compared to Cd-treated groups, in host and water microbial communities at T1 and T3, especially when the low abundant OTUs were discarded from the NLS models (see the neutral OTUs percentage and goodness of fit across OTUs abundance cut-offs, Figs. 1e and 2e, Supplementary Figure 9, Supplementary Table 6).

**Fig. 7:**
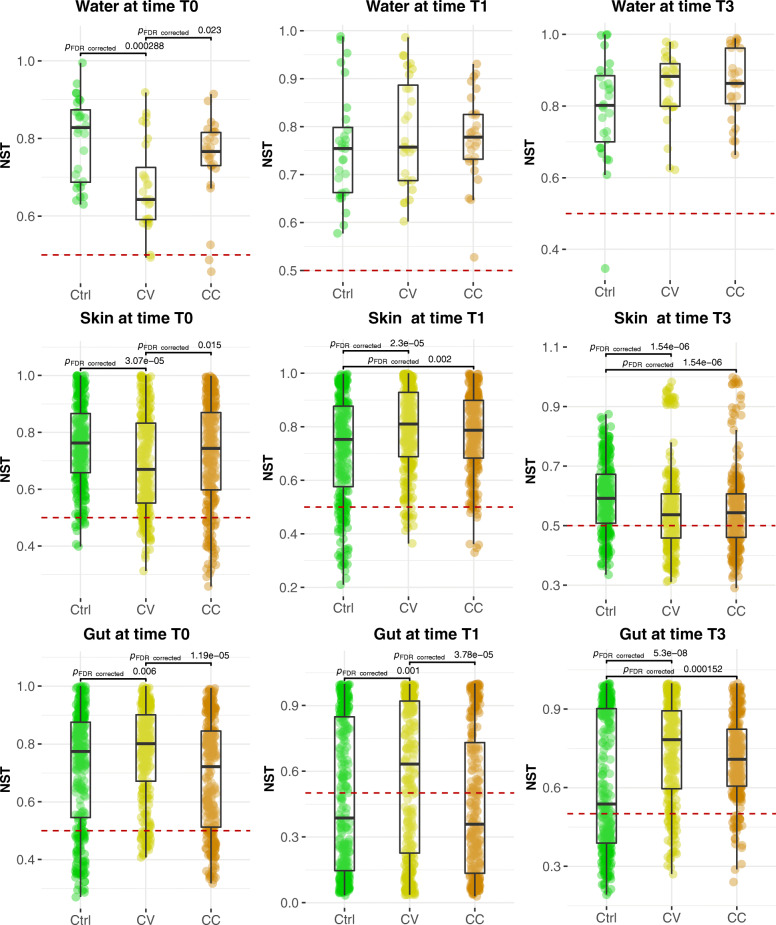
Comparative analysis of stochasticity ratios averages between treatments. The NST values were computed using the NST model available in the R package. With this model the” Proportional fixed” null-model, Jaccard similarity coefficient (also known then as Ruzicka similarity) and *n* = 1000 for random permutations were used to compute the normalized stochasticity ratios. The NST in each group represented in violin plots were compared for their average using Kruskal-Wallis test followed by Benjamini-Hochberg as a multiple correction test. Only the significant Dunn test *p*-values for pairwise comparisons are displayed on this figure, however in order to improve visibility, the significant *p*-values of Kruskal-wallis test for multiple groups comparisons were not plotted

To quantify the ecological stochasticity in the community structure, we applied a null modelling approach using normalized stochastic ratio (NST) index (see Materials and methods). When NST is higher than 0.5, the community structure is more likely driven by the neutral processes, whilst when the NST is lower, the main driving processes are deterministic. According to the results, at each time point, and in all treatments, the average of NST (NST_avg._) for almost all communities is higher than 0.5 (Fig. 7). Comparing stochasticity ratios between water and host microbiota, the NST modelling lead to same results of NLS models. The NST_avg._ values indicate stochasticity equal (at time T0) or higher (at T1 and T3) in water compared to the gut and skin microbiota. When it comes to comparing the magnitude of stochasticity between treatments and control in each community type structure, both NST and NLS models agree in terms of the dominance of neutral processes in the community assembly (goodness of fit R^2^ > 0.5), and structure (NST_avg_ > 0.5), although giving further insights in both demographical and ecological aspects, respectively. With NLS model, the percentage of neutral OTUs and the goodness of fit are always higher in the control group, suggesting that a higher proportion of non-neutral OTUs influenced demographical dynamics in Cd-treated groups relatively to control group. With NST models, the ecological stochasticity in the community structure was significantly higher (NST_avg_ > 0.5) that the null hypothesis in all groups. Furthermore, higher averages of ecological stochasticity were observed under perturbations in Cd-treated groups early at T1 in the skin microbiome and lately at T3 in the gut microbiome (Fig. 7).

## Discussion

Our study evidenced salient differential changes in community assembly across three community types (environmental or host-associated), time and xenobiotic exposure regimes. This study for the first time highlights the relative contribution of neutral and non-neutral factors in shaping the microbiota during the early life-stages of an ecotoxicology vertebrate model. First, alpha diversity increased significantly in the skin and gut microbiota in both constant and gradual selection regimes. Then, at the community-level, significant phylogenetic divergence was observed between the control and treatment groups in the three community types at two specific time points, T1 and T3. These two key time points were investigated further with co-abundance network analysis and community assembly modelling. Frequent significant negative correlations between taxa in both selection regimes in the skin and the increasing richness of environmental bacterial strains suggest a dysbiosis in the mucosal host-associated microbiota [64, 65]. Negative correlations between OTUs occur is all groups at time T1, but they are remarkably more frequent in both selection regimes within the skin microbiome networks. In many studies, the increase of negative correlations in networks was associated to microbiota dysbiosis in several contexts of human lung infectious diseases [66–68], in dog’s gut (Vázquez-Baeza et al. [69]) and macaque model of tuberculosis for instance. Furthermore, network connectivity under stress was maintained by rare OTUs, while abundant OTUs were mainly composed of opportunistic invaders such as *Mycoplasma* and other genera related to fish pathogens like *Aeromonas, Pseudomonas* and *Flavobacterium* [70, 71].

Lastly, to predict the nature of evolutionary processes driving the metacommunity assembly under Cd perturbations, the fit of the neutral model, based non-linear least squares (NLS), indicates that neutral processes predominantly drove taxonomic drift during the assembly of host and water microbial communities in all experimental groups (*R*^*2*^ > 0.5). However, regarding the percentage of neutral OTUs between groups suggests significantly lower stochasticity of community assembly in Cd-treated groups. At this point, from the higher percentage of non-neutral OTUs, we propose that Cadmium perturbations have an effect not only on the community assembly but also on the community structure. Previous studies quantified high ecological stochasticity in the community structure under perturbations by suggesting specific [72] and general indexes [62] of stochastic strength. The application of NST model to our data (NST_avg_ > 0.5) confirms high ecological stochasticity in the community structure changes under disturbance, as suggested by Ning and co-authors [62], and this result agrees with the dysbiosis and the model of resistance to colonisation. However, we disagree with the Ning et al. (2019) [62] on the comparison of the NLS model with NST indexes, because this comparison is meaningless. The general NST null model was developed for quantitatively assessing ecological stochasticity, while the NLS model Sloan’s neutral model was developed for quantitatively assessing the demographic stochasticity. Initially, the NST model was developed and applied to estimate the stochasticity strength during the succession of a groundwater microbial community in response to perturbations caused by organic carbon (vegetable oil) injection [62]. The authors of NST have also compared their model index with not normalized stochasticity strength measure (an early version of NST) [72] and the percentage of neutral OTUs predicted by the NLS of Sloan’ model [22]. NST demonstrated high stochasticity under perturbation (oil injection), and in our host-microbiome system, we found the same trends. On the other side, the NLS model has different assumptions based on the estimation of the emigration rates (can be interpreted as dispersal limitation index) from a community source but not based on an ecological similarity/dissimilarity distance. To our knowledge, with the NST model, we could not distinguish between neutral and non-neutral OTUs or quantify a migration rate to assess the dispersal ability from the community structure. From our point of view, the comparison of NST with NLS could not infer the relative contribution of deterministic processes. The NST authors ‘model also acknowledged in their discussion that the operational distinction of stochasticity and determinism can appear somewhat arbitrary with their NST index, and it is difficult to distinguish ecological stochasticity from the noise caused by deterministic environmental factors, as shown in their simulation [62].

Last not least, we would stress that NLS model, with our null hypothesis based on a real bacterial community (i.e. the control community), detected non-negligible proportion of non-neutral OTUs, potentially suggesting that host-microbiota assembly partly resulted from deterministic processes. Although moderate, the percentage of OTUs not fitting the NLS model in both Cd regimes may suggest patterns of deterministic effects on microbiota recruitment [27] host filtering and Cd treatment. The host filtering can be interpreted lately at T3 throughout the network’s modularity and average connectivity in the control group. The high modularity and degree observed at T3 would approach specialized, partitioned microbiome networks in the gut and skin [73, 74], reflecting niche differentiation [75] and microbiota maturation along with perch juvenile’s development [3]. However, in an opposite direction, the gut and skin networks modularity and degree decreased over time to reach a lower average in CC & CV groups, suggesting broader niche partitioning [74] and lesser interactions and more stochastic changes under disturbance [62].

The dominant ecological stochasticity quantified under disturbance left interesting patterns in the community structure of the skin microbiota. At T1, significant phylogenetic divergence occurred between treatments (CC & CV) and control (Control). Most importantly, a taxonomic convergence between treatments (CC & CV) not only in the skin but also within the water community occurred. This convergence mainly resulted from an invasion of environmental bacterial strains in the skin. The significant increase of Bacteroidetes only in treatments groups (CC &CV) in skin communities compared to water communities could strongly support this hypothesis. Such gain or loss of tissue-specific community type suggests a disruption of the host’s ability to control the assembly of skin microbiota, which is correlated with Cd exposure. This phenomenon is termed “direct colonisation resistance” [76]; however, we do not exclude that this colonisation failure also resulted, at least partly, from a host immune system failure (termed as “immune colonisation resistance”). This compositional disruption translated into many negative correlations between taxa in both selection regimes in the skin-associated microbiota at T1. Furthermore, the impact of Cd exposure on skin community structure was also observed at T3, where the phylogenetic distance became significantly divergent, even between both selection regimes, where many negative correlations were detected between taxa. In addition to the increasing invasion of environmental bacterial strains in the skin (i.e. failure of colonisation resistance, see [76]), and the rise of negative correlations suggest a dysbiosis state of skin-associated microbiota [66–69, 77]. This dysbiosis might be associated not only with an increase in evenness and phylogenetic convergence with the water bacterial community but also with the rise of antagonism among OTU co-abundance networks in both selection regimes (CC & CV). Most of antagonism was mediated through rare and abundant Tenericutes *(Mycoplasma)* and Proteobacteria. Depending upon the strain, Mycoplasmas are thought to be either fish opportunists, or innocuous commensals in fish [78, 79]. In comparison to the skin, the significant divergence between control (Control) and treatments (CC, CV), and the rise of negative correlations, appeared later in the gut community: at T3. This delayed pattern of dysbiosis strongly suggests that the physiological impact of cadmium exposure was mitigated more effectively within the gut. In fish (and other vertebrates), the liver is the main organ to accumulate xenobiotics including cadmium. Therefore, the late compositional change in the gut microbiota potentially occurred when bioaccumulation of cadmium within the liver reached its maximum carrying capacity. Another noticeable compositional change was the disconnection of abundant taxa from the main gut interacting network, which was proportional to the stress intensity. By T3, the overall taxonomic network connectivity was formed exclusively from rare OTUs. Contrastingly, abundant OTUs were peripherals and disconnected from central hubs [63], mainly composed of putative opportunistic invaders such as *Mycoplasma* and other genera encompassing strains associated to fish pathogens, like *Bacillus* (> 6% in CC and CV). As observed in other fish species such as Atlantic salmon (*Salmo salar*) [80] and the long jaw mudsucker (*Gillichythys mirabilis*) [81, 82], Yellow Perch have intestinal microflora dominated by Tenericutes (*Mycoplasma sp*.). It is therefore difficult to conclude whether the increase of several *Mycoplasma* strains is beneficial or not to the host. Concerning *Bacillus,* a similar increase was associated with irritable bowel disease (IBD) in dogs and negatively correlated with bacterial strains associated with healthy individuals [69]. Interestingly, rare OTUs and negative correlations did not play an essential role in water co-abundance networks, which were highly fragmented in Cd treatment regimes, and the average connectivity was significantly higher in the control regime. It is worthy to notice that the taxonomic composition of water bacterial communities was characterized by a very low occurrence of Tenericutes (*Mycoplasma*) compared to host communities. The metacommunity networks combining host and water bacterial communities highlights the niche divergence and dynamics between the water bacterial communities and each of the host microbiota. A T0, all these community types showed interactions within a distinctive structure of subnetworks, which differentiated over time in independent hubs weakly connected (better connected in the control group).

Finally, low abundant or rare OTUs have been demonstrated to play a pivotal role in community assembly [35–39] either in promoting homeostasis [37] or dysbiosis [83]. Therefore, we applied the NLS model to disentangle neutral and non-neutral evolutionary processes that were at play for rare OTUs. The variation of the presence of neutral OTUs across different abundance cut-offs (Supplementary Table 6) suggested a neutral role around 35 to 50% of rare OTUs in host water communities. Overall, our data demonstrated that stochastic processes mainly drove taxonomic drift even under Cd disturbance. However, host-microbiota assembly evolved by involving non-neutral processes in cadmium treatment groups, although this trend was less salient in both experimental groups at T3. The majority of the OTUs that did not fit the neutral model was assigned to *Mycoplasma* genus under and after the exposure during the recovery period [40]. The assembly of gut microbial communities may have evolved under non-neutral processes due not only to the cadmium as a disrupting factor but also due to the selection imposed by the host development [22].

## Conclusions

In this experimental evolution study, our findings demonstrate the extensive involvement of low abundant (rare) taxa throughout community assembly and interacting network connectivity under perturbations. We have unearthed a niche-specific response to cadmium disturbance. In the skin microbiome network, we detected early signals of antagonistic interactions by a preponderance of negative correlations, whilst at the same time, the overall connectivity in the gut microbiome was degraded over time. The network topology in the control group suggests specialized, partitioned microbiome interactions in the gut and skin, reflecting niche differentiation and microbiota maturation along with perch juveniles’ development. On the other hand, the network topology for Cadmium-treated groups suggests a broader niche, fewer interactions. Considering the modelling frameworks, neutral processes were thus the major forces of the community assembly in the environmental microbiome, although involving a low percentage of deterministic changes in the host-microbiota under disturbance. The taxonomic convergence between water- and skin-associated bacterial communities across both cadmium exposure groups highlights the loss of the colonization resistance capacity of the host. This was due to physiological stress experienced by the host: cadmium bioaccumulation in Perch’s liver has already been documented to disrupt host physiology. By highlighting the link between a loss of colonization resistance and dysbiosis within the host (which in turn is known to induce an inflammatory response), our results will be useful not only for the field of microbial ecology but also for biomedical research, as dysbiosis of gut microbiome composition has been shown to result in the onset of various inflammatory diseases such as diabetes, IBD, Crohn disease, cancer, and obesity [84–86].

### Perspective

The patterns of neutral and non-neutral assembly in contrasting types of bacterial communities (i.e. one environmental and two host-associated) described here provide novel key insights regarding our understanding of evolutionary forces that are at play in shaping the host-microbiota when facing sublethal environmental stress. Living organisms are currently facing unprecedented levels of environmental stressors that impact their capacity to cope with natural pathogens, essentially by altering their overall immune defence. Therefore, there is an urgent need to accurately decipher the early warning signals occurring at the first stages of xenobiotic exposure.

## Supplementary Information

**Additional file 1: Table S1.** ANOVA summary of metals concentrations in water tanks and *Perca flavescens* fish livers. Tukey and Wilcoxon’s tests showed that the Cadmium concentration in fish liver and water has significantly changed between treatments and Control; Constant Cadmium regime (CC), Variable Cadmium regime (CV), and Control (Control) and overtime. No significant changes were observed in Zinc and Cooper between treatments.

**Additional file 2: Table S2.** Statistical summary of alpha-diversity changes over time and treatments. Over time, the richness and evenness have significantly changed in treatments and Control for all types of communities. The significant changes of alpha-diversity between treatments and Control were statistically demonstrated (Table 2b) using rank statistics tests (Kruskal-Wallis/Wilcoxon). The same statistics were used to compare alpha-diversity overtime. Overall, the evenness in microbial skin communities has significantly diverged between treatments and Control at T1, and richness in Gut microbial communities at T3.

**Additional file 3: Table S3.** Phylogenetic divergence in host and water microbiomes. The phylogenetic distances between OTUs were computed using Gunifrac distance (see Materials and Methods). The divergence between treatments and control was assessed using PERMANOVA, and the homogeneity for group dispersions (distance from centroid) was evaluated using two multivariate tests, BETADISPER and Multi-Response Permutation Procedure (MRRP) of within versus among group dissimilarities. The significance of divergence between groups was measured by applying multiple correction tests with Benjamini-Hochberg BH (*p*-value< 0.05).

**Additional file 4: Table S4.** Summary of correlation network features in host and water microbial communities. The networks of gut microbial communities are composed of 30, 46, 65 of CC (connected components or hubs), respectively in Control, CV, and CC groups (see sheet 1). Therefore, the nodes number (NN) was lower in the Control group (Control_NN_: 410) compared to Cd regimes networks (CV_NN_:488; CC_NN_:526). On the contrary, an average of neighbours (AN) was higher in the Control network (Control _AN_: 4) than in Cd groups networks (CV_AN_:2.98; CC_AN_:2.82). Skin microbial networks were composed of 47, 10, 3 of CC (connected components), respectively in Control, CV, and CC groups (see sheet 1). The number of CC was inversely proportional to nodes number, and low in Control group network (Control_NN_: 651) in comparison with Cd networks (CV _NN_: 661; CC _NN_:759). Similarly, the average of neighbours (AN) was lower in the Control network (Control _AN_: 4) compared to Cd group networks (CV_AN_: 10.7; CC_AN_: 24.6). This high connectivity in CV and CC at T1 was proportional to the high percentage of strong negative correlations (R neg. Corr. < − 0.6; B-H *p*-value < 0.05), observed in those groups (CV neg. Corr.: 6.3%; CC neg. Corr.: 6.9%) compared to Control group (Control neg. Corr.: 2.88%). Water microbial networks indicate variables features over time and between regimes (see sheet 2).

**Additional file 5: Table S5.** Significant taxonomic changes in water and host-microbial communities over time and between treatments. In the gut, the significant changes in the relative abundance between T0 and T1 were detected for Synergistetes in CC and Tenericutes in CC and CV. Later, between T1 and T3, the relative abundance of four phyla, Euryarchaeota, Firmicutes, Proteobacteria, and Tenericutes has significantly changed in all groups. In the skin, between T0 and T1, the significant changes were observed for Firmicutes and Fibrobacteres in the CV, and Bacteroidetes and Fusobacteria in CC and CV. However, between T1 and T3, the significant changes of relative abundance concerned Actinobacteria in CC and CV; Proteobacteria, Tenericutes, Bacteroidetes, and Firmicutes in all groups. In water, between T0 and T1, the significant changes were observed for Bacteroidetes in Control; Proteobacteria in the CV; Firmicutes and Tenericutes in all groups. However, between T1 and T3, the significant changes concerned Proteobacteria and Tenericutes in CV, and Firmicutes in all groups. This table only summarized significant taxa variation with multiple test corrections (Benjamini-Hochberg p-value BH < 0.05) performed on significant Wilcoxon rank-sum test.

**Additional file 6: Table S6.** This table summarises the variation of the goodness of fit (R^2^) to the neutral model, the percentage of neutral and non-neutral OTUs through different abundance cut-offs in the different treatment groups of host and water microbial communities at times T1 and T3.

**Additional file 7: Figure S1.** Box plots of alpha-diversity variations over time and between treatments in the host and water microbial communities. The boxplots of richness and evenness variations showed different trends between treatments and Control. In the gut, the alpha-diversity showed the same tendency in all groups, except at time T3. In the skin, the evenness at T1 was higher in Cadmium treatments compared to Control while the opposite produced for richness at T3. In water, the evenness and richness were intermediate in the Control group compared to variable and constant Cadmium selection treatments, except for the evenness which was the highest in the Control group at T1. Constant Cadmium regime (CC) is in orange, variable Cadmium regime (CV) is in Yellow, and Control (Ctrl) is in green.

**Additional file 8: Figure S2.** Heatmaps of significant taxonomic variation at the phylum level. This figure includes three heatmaps representing significant overtime changes of taxonomic composition at the phylum level in the gut (2a.), skin (2b.) and water (2c.). The hierarchical clustering of the relative abundance of phyla which significantly changed over time was performed using Ward’s method and Bray–Curtis dissimilarity distance. Vegan package and heatmap () function in R were used to produce these heatmaps.

**Additional file 9: Figure S3.** Heatmaps of significant taxonomic variation at the genus level. This figure indicates with three heatmaps the significant overtime changes of taxonomic composition at the genus Level in GMC (Gut Microbial Community) (2a.), SMC (Skin Microbial Community) (2b.) and WMC (Water Microbial Community) (2c.). The hierarchical clustering of the relative abundance of phyla, which significantly changed over time was performed using Ward’s method and Bray–Curtis dissimilarity distance. Vegan package and heatmap () function in R were used to produce these heatmaps.

**Additional file 10: Figure S4.** Phylogenetic divergence at the community-level in the water microbiome. NMDS (non-metric Multi-Dimensional Scaling) plot of generalized Unifrac distances showing the distribution of the water samples based on the phylogenetic content of their microbiota. The plot shows a significant separation of sample groups according to treatment (see in Supplementary Table 5-b, *p*-values of the PERMANOVA test indicating the significance of group separations) at each time point, but treatment samples are closer to each other than the Control group at T1 and T3.

**Additional file 11: Figure S5.** Boxplots of Bacteroidetes variation over time and between treatments. At T0, the relative abundance of Bacteroidetes was significantly lower in the skin compared to the water and the gut microbial communities. However, at T1, Bacteroidetes abundance was significantly higher (Wilcoxon ‘s *P*-value < 0.05) in the skin microbial communities only in treatment groups (CC and CV), while in the Ctrl group they showed any significant variation.

**Additional file 12: Figure S6**. Water and host-microbial interactions network over time and between communities. This figure summarises the dynamic of interactions of water with the host microbiome networks. Each node size in the network is proportional to the average of the OTUs relative abundance in all samples. These networks are based on significant Spearman coefficients and were constructed using R scripts and Cytoscape software.

**Additional file 13: Figure S7**. Statistical analysis of the closeness centrality in the water and host-microbiome networks. The comparison of centrality assessed with nodes closeness centrality and represented with violin plots indicate that the average of connections is significantly higher in the skin compared to the water and gut microbiomes in all treatments at T0 before disturbance. However, at T1 the centrality of node converged (which means not significantly different) between the water and skin microbiome for cadmium-treated groups. At T3, the centrality of node converged between the gut and skin microbiome in the control group. The average of nodes’ degree computed with Network Analyzer was compared using the Kruskal-Wallis test followed by Benjamini-Hochberg test. The value of 0.05 is the threshold of B-H p-value significance.

**Additional file 14: Figure S8**. Skin microbiome networks built with SPIEC method. The SPIEC-EASI (SParse Inverse Covariance Estimation for Ecological Association Inference) method [55] implemented in R was applied using Meinshausen-Buhlmann’s neighbourhood selection (MB) method to estimate the inverse covariance matrix. The OTUs having low frequency occurrence (occurrence <=3) were dummied in one synthetic OTUs (black node). Red and grey edges represent negative and positive regression coefficients of the inverse covariance matrix. The node size in the network is proportional to the average of an OTU relative abundance in all samples. These networks were visualized using Cytoscape software.

**Additional file 15: Figure S9.** Distribution of neutrality versus abundance cut-off and goodness of fit. This figure shows the variation of neutral OTUs percentage (Y-axis) and goodness of fit predicted by NLS models using 12 cut-offs thresholds of relative abundance percentages (gg facet panels) in the entire metacommunity at T1 and T3.

**Additional file 16: Supplementary file 1**.

**Additional file 17: Supplementary file 2**.

**Additional file 18: Supplementary file 3**.

## Data Availability

Sequencing data are available in the Sequence Read Archive (SRA) database at NCBI under the BioProject ID PRJNA556617.

## Abbreviations

ANOVA: Analysis of variance
16 s rDNA: 16S Ribosomal DNA
BEB: Back extraction buffer
BH: Benjamini-Hochberg correction test
CdCl2: Cadmium Salts
CPAUL: Comités de Protection des Animaux de l’ Université Laval
Ctrl: The regime of negative Control
CV: The regime of Cadmium Variable Concentration
CC: The regime of Cadmium Constant Concentration
IBD: Irritable bowel disease
m: Emigration rate
m.mle: Maximum likelihood estimation of emigration rate
NLS: Non-linear least-squares model
NST: Normalized stochasticity ratios (NST)
MRPP: Multiple Response Permutation Procedure
NMDS: Non-metric Multi-Dimensional Scaling
OTUs: Operational Taxonomic Units
ppb: Parts per billion
PCoA: Principal Coordinates Analysis
PCR: Polymerase chain reaction
PERMANOVA: Permutational analysis of variance
RDP: Ribosomal Database Project
R^2^: Goodness of fit
