# Supplementary material for: The yellow perch (Perca flavescens) microbiome revealed resistance to colonisation mostly associated with neutralism driven by rare taxa under cadmium disturbance
Source: Anim Microbiome. 2021 Jan 5;3:3. doi: 10.1186/s42523-020-00063-3 (PMC7934398; doi:10.1186/s42523-020-00063-3)
Supplement: Supplementary file 7 — Additional file 7: Figure S1. Box plots of alpha-diversity variations over time and between treatments in the host and water microbial communities. The boxplots of richness and evenness variations showed different trends between treatments and Control. In the gut, the alpha-diversity showed the same tendency in all groups, except at time T3. In the skin, the evenness at T1 was higher in Cadmium treatments compared to Control while the opposite produced for richness at T3. In water, the evenness and richness were intermediate in the Control group compared to variable and constant Cadmium selection treatments, except for the evenness which was the highest in the Control group at T1. Constant Cadmium regime (CC) is in orange, variable Cadmium regime (CV) is in Yellow, and Control (Ctrl) is in green. [file 42523_2020_63_MOESM7_ESM.pdf]

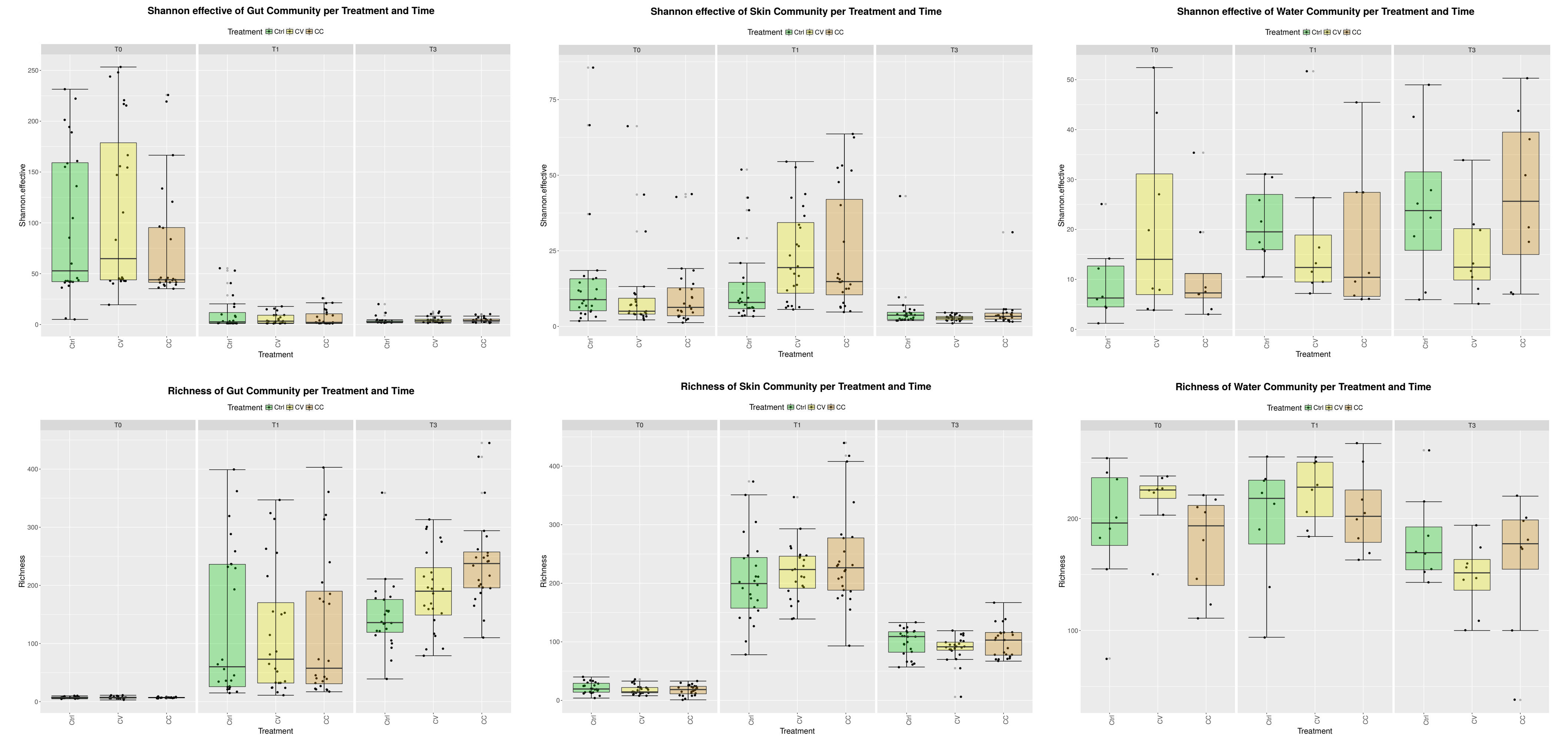
Shannon effective of Gut Community per Treatment and Time
Shannon effective of Skin Community per Treatment and Time
Shannon effective of Water Community per Treatment and Time
Richness of Gut Community per Treatment and Time
Richness of Skin Community per Treatment and Time
Richness of Water Community per Treatment and Time
Treatment Ctrl CV CC
T0
T1
T3
Shannon.effective
Richness
Treatment
Ctrl
CV
CC
