# Supplementary material for: The yellow perch (Perca flavescens) microbiome revealed resistance to colonisation mostly associated with neutralism driven by rare taxa under cadmium disturbance
Source: Anim Microbiome. 2021 Jan 5;3:3. doi: 10.1186/s42523-020-00063-3 (PMC7934398; doi:10.1186/s42523-020-00063-3)

S.2A-Significant overtime variation of the Gut taxa at the phylum−level

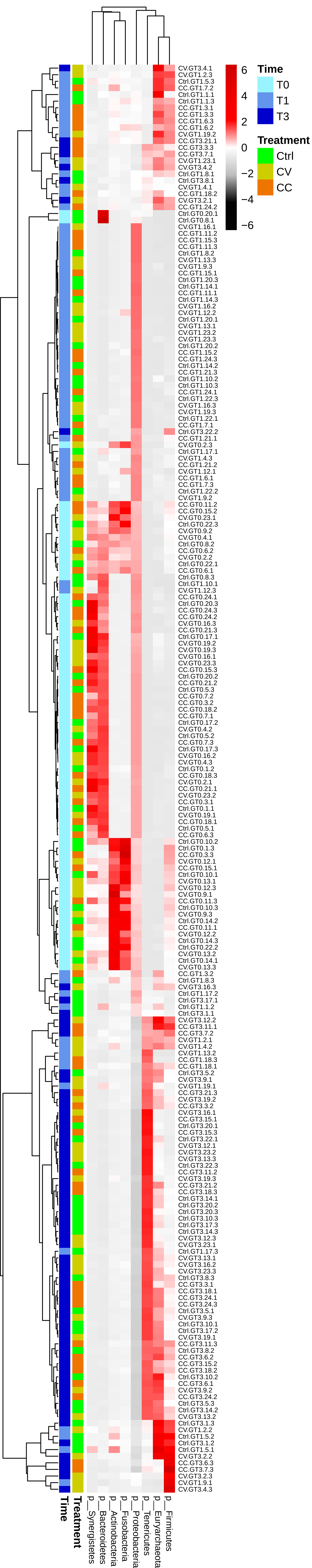

S.2B-Significant overtime variation of the Skin taxa at the phylum−level

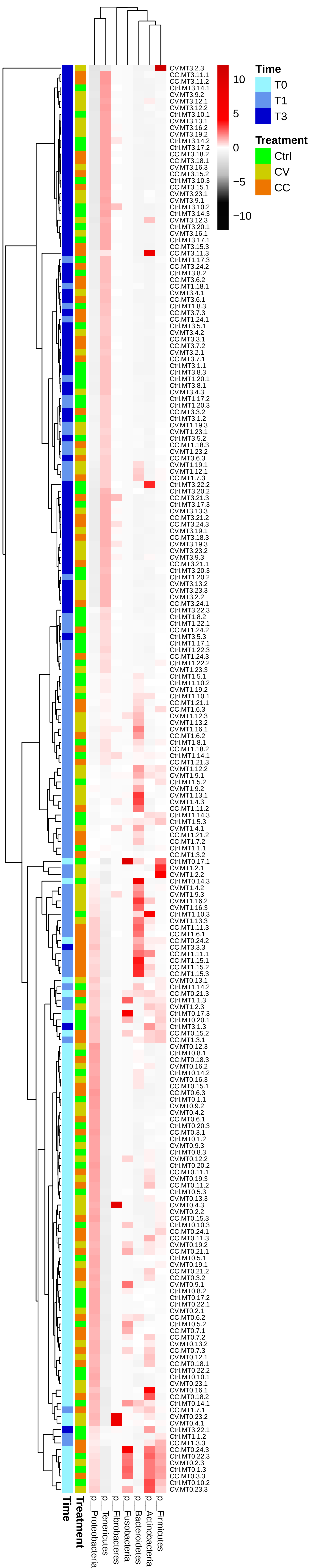

S.2C-Significant overtime variation of the Water taxa at the phylum−level

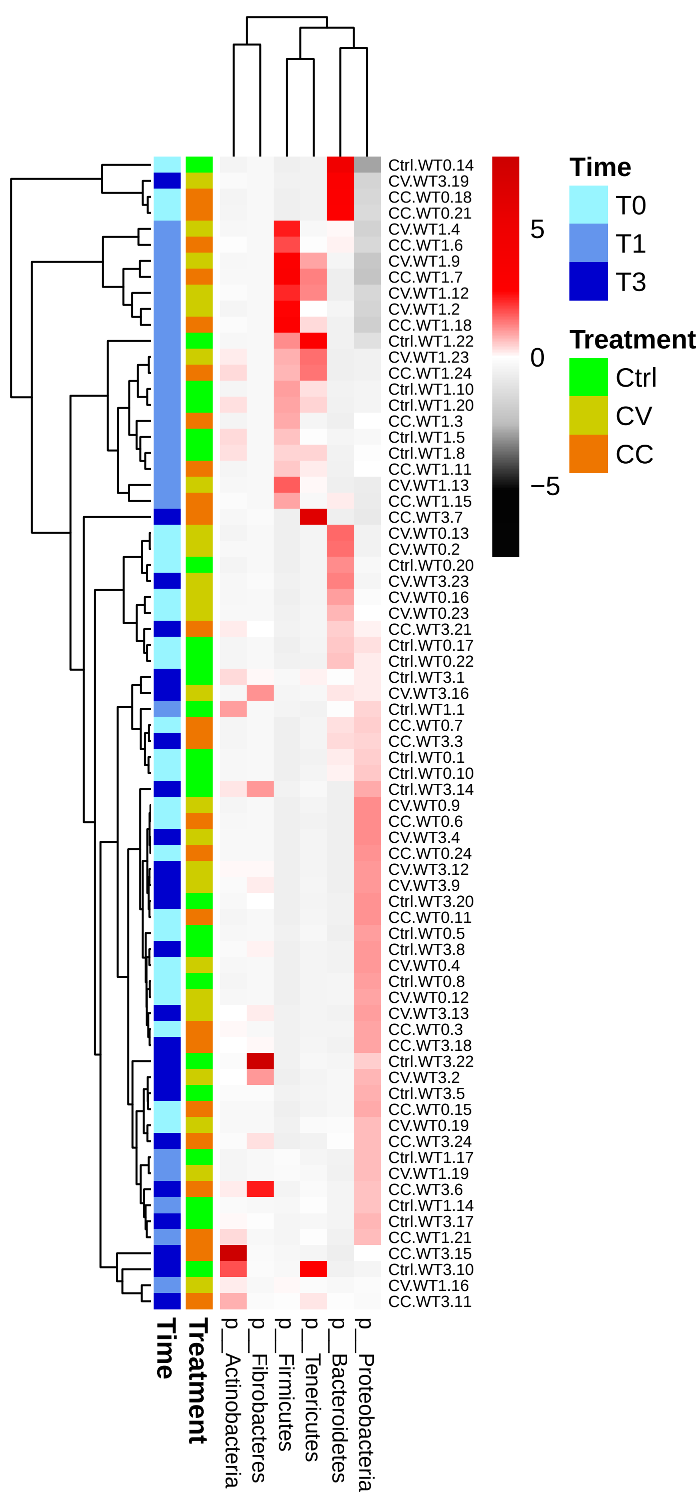

Supplement: Supplementary file 8 — Additional file 8: Figure S2. Heatmaps of significant taxonomic variation at the phylum level. This figure includes three heatmaps representing significant overtime changes of taxonomic composition at the phylum level in the gut (2a.), skin (2b.) and water (2c.). The hierarchical clustering of the relative abundance of phyla which significantly changed over time was performed using Ward’s method and Bray–Curtis dissimilarity distance. Vegan package and heatmap () function in R were used to produce these heatmaps. [file 42523_2020_63_MOESM8_ESM.pdf]
