# Supplementary material for: The yellow perch (Perca flavescens) microbiome revealed resistance to colonisation mostly associated with neutralism driven by rare taxa under cadmium disturbance
Source: Anim Microbiome. 2021 Jan 5;3:3. doi: 10.1186/s42523-020-00063-3 (PMC7934398; doi:10.1186/s42523-020-00063-3)

S.3A-Significant overtime variation
of the Gut taxa at the genus−level

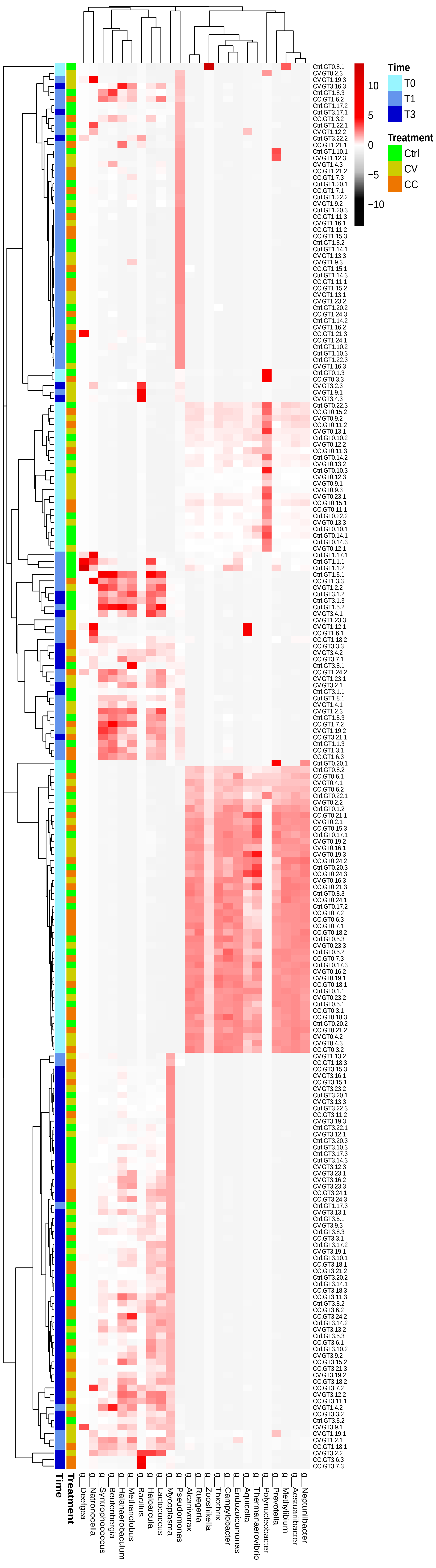

S.3B-Significant overtime variation
of the Skin taxa at the genus−level

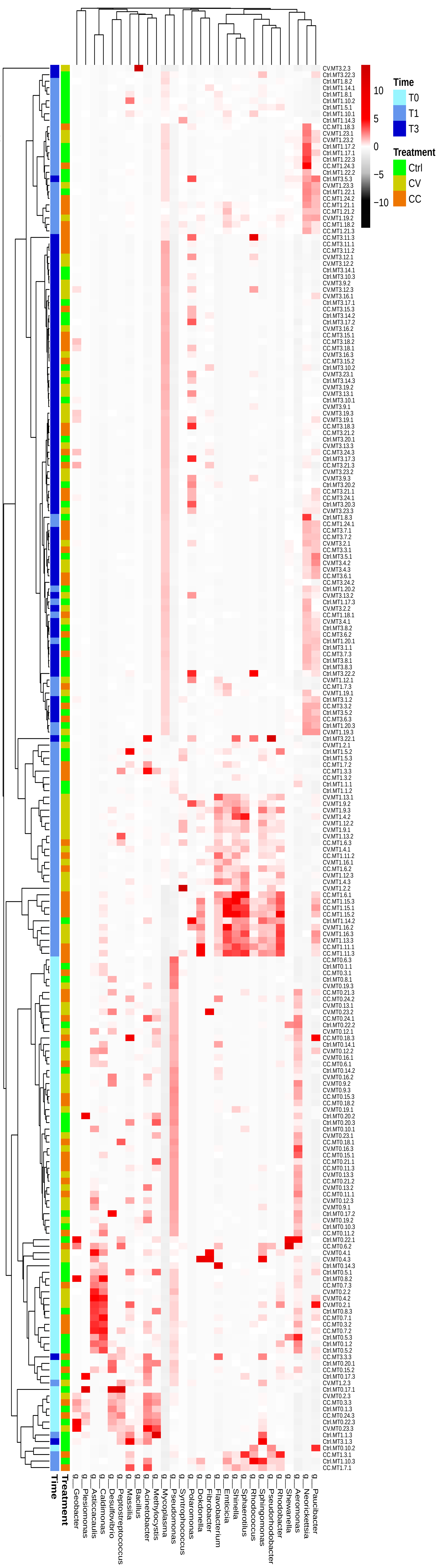

S.3C-Significant overtime variation
of the Water taxa at the genus−level

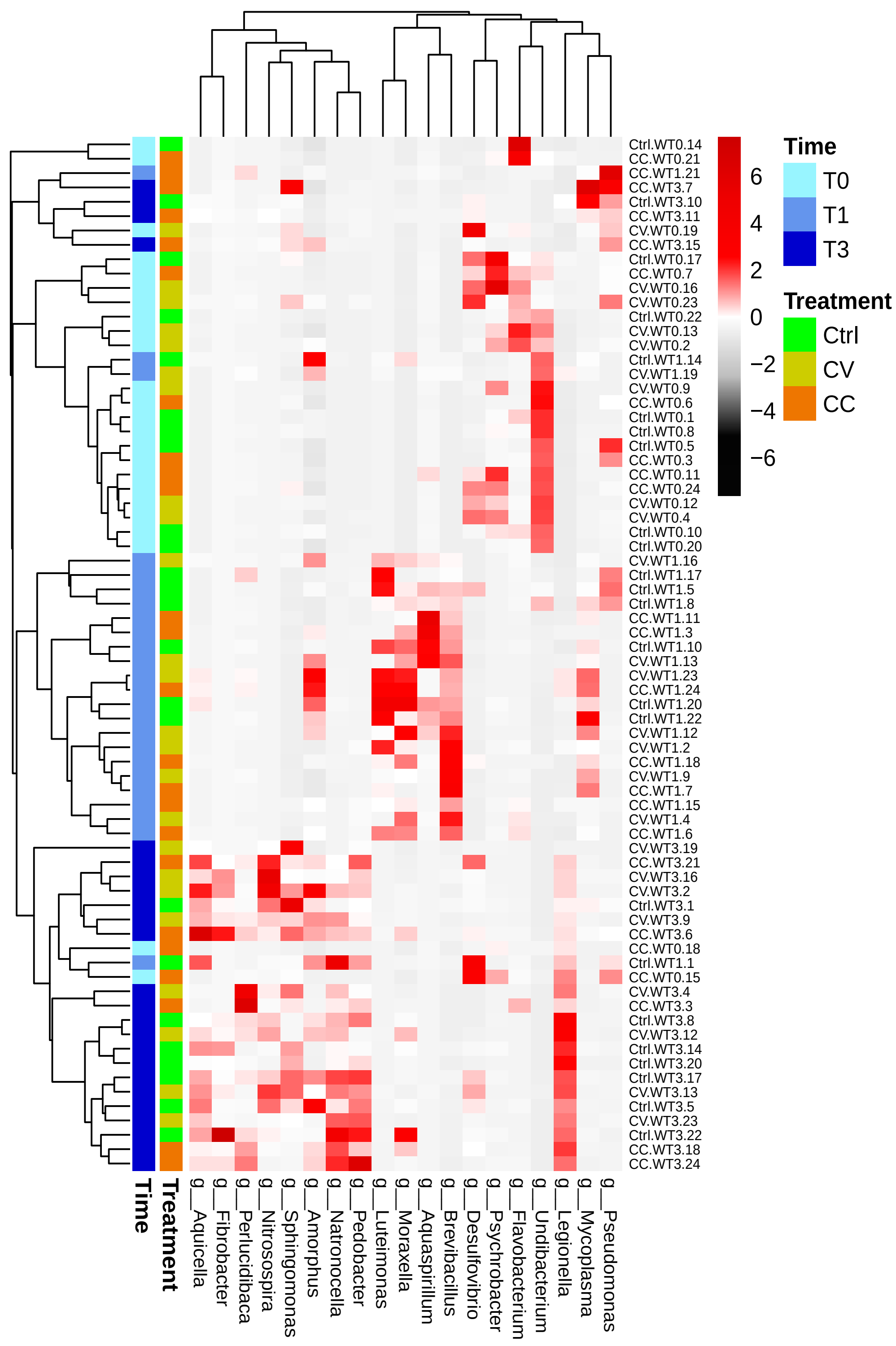

Supplement: Supplementary file 9 — Additional file 9: Figure S3. Heatmaps of significant taxonomic variation at the genus level. This figure indicates with three heatmaps the significant overtime changes of taxonomic composition at the genus Level in GMC (Gut Microbial Community) (2a.), SMC (Skin Microbial Community) (2b.) and WMC (Water Microbial Community) (2c.). The hierarchical clustering of the relative abundance of phyla, which significantly changed over time was performed using Ward’s method and Bray–Curtis dissimilarity distance. Vegan package and heatmap () function in R were used to produce these heatmaps. [file 42523_2020_63_MOESM9_ESM.pdf]
