# Supplementary figures and images for: The yellow perch (Perca flavescens) microbiome revealed resistance to colonisation mostly associated with neutralism driven by rare taxa under cadmium disturbance
Source: Anim Microbiome. 2021 Jan 5;3:3. doi: 10.1186/s42523-020-00063-3 (PMC7934398; doi:10.1186/s42523-020-00063-3)

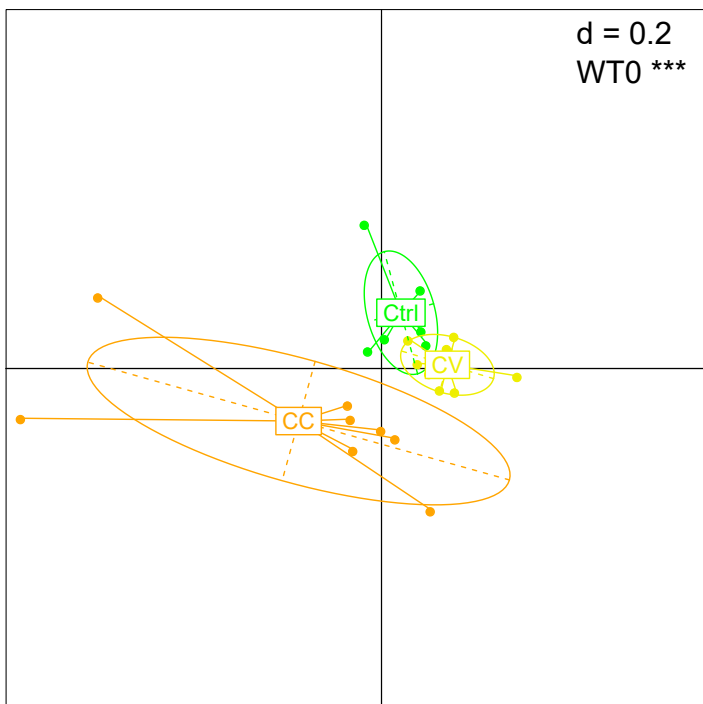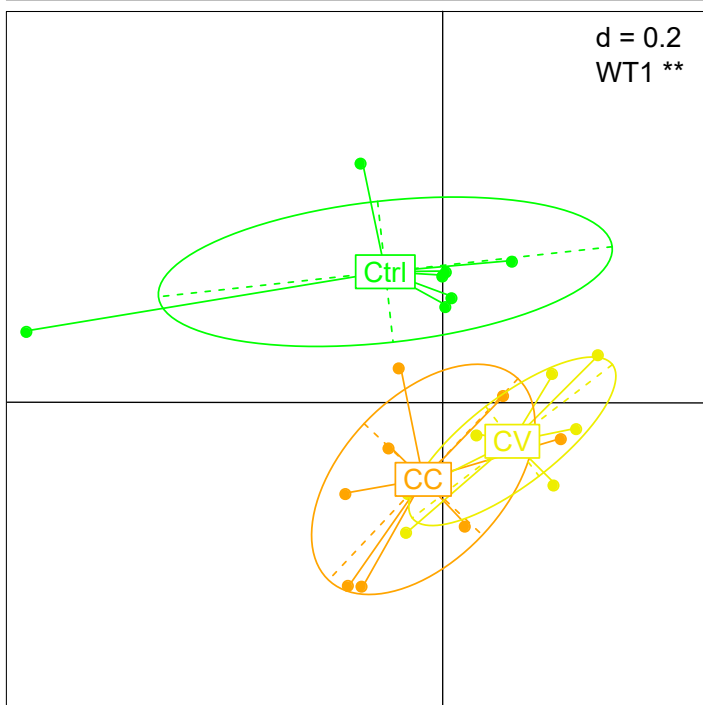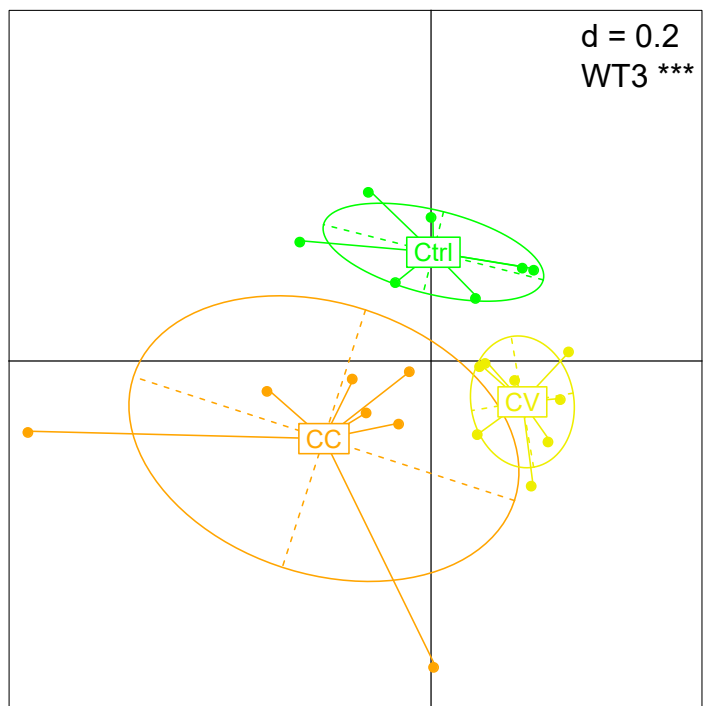

Supplement: Supplementary file 10 — Additional file 10: Figure S4. Phylogenetic divergence at the community-level in the water microbiome. NMDS (non-metric Multi-Dimensional Scaling) plot of generalized Unifrac distances showing the distribution of the water samples based on the phylogenetic content of their microbiota. The plot shows a significant separation of sample groups according to treatment (see in Supplementary Table 5-b, p-values of the PERMANOVA test indicating the significance of group separations) at each time point, but treatment samples are closer to each other than the Control group at T1 and T3. [file 42523_2020_63_MOESM10_ESM.pdf]
