# Supplementary material for: The yellow perch (Perca flavescens) microbiome revealed resistance to colonisation mostly associated with neutralism driven by rare taxa under cadmium disturbance
Source: Anim Microbiome. 2021 Jan 5;3:3. doi: 10.1186/s42523-020-00063-3 (PMC7934398; doi:10.1186/s42523-020-00063-3)
Supplement: Supplementary file 11 — Additional file 11: Figure S5. Boxplots of Bacteroidetes variation over time and between treatments. At T0, the relative abundance of Bacteroidetes was significantly lower in the skin compared to the water and the gut microbial communities. However, at T1, Bacteroidetes abundance was significantly higher (Wilcoxon ‘s P-value < 0.05) in the skin microbial communities only in treatment groups (CC and CV), while in the Ctrl group they showed any significant variation. [file 42523_2020_63_MOESM11_ESM.pdf]

T0

Gut : G ; Skin mucus : M; Water : W

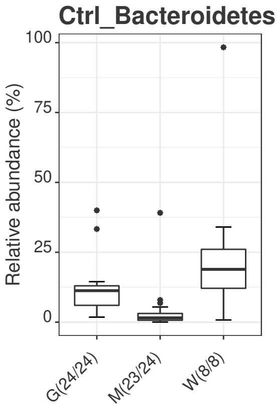

Wilcoxon Rank Sum Test – pairwise

Groups	P–value	Adj. p–value
1–2	0.0000	0.0000
2–3	0.0015	0.0023

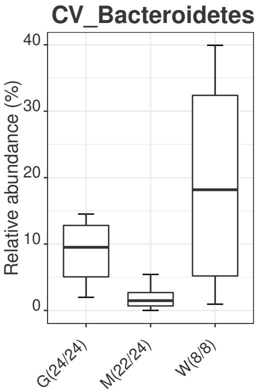

Wilcoxon Rank Sum Test – pairwise

Groups	P–value	Adj. p–value
1–2	0e+00	0e+00
2–3	3e–04	4e–04

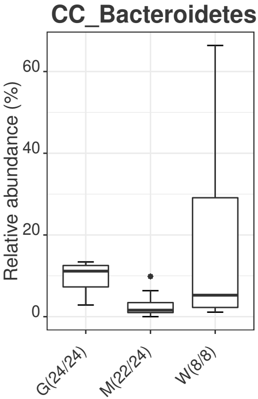

Wilcoxon Rank Sum Test – pairwise

Groups	P–value	Adj. p–value
1–2	0.0000	0.0000
2–3	0.0308	0.0462

Gut : G ; Skin mucus : M; Water : W

T1

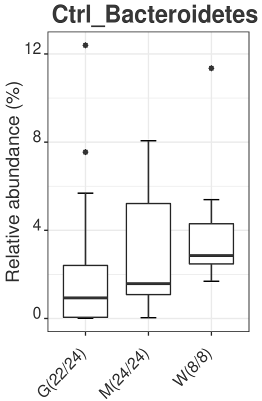

Wilcoxon Rank Sum Test – pairwise

Groups	P–value	Adj. p–value
1–3	0.027	0.081

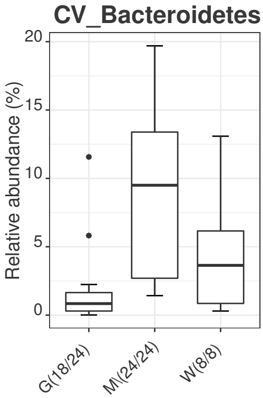

Wilcoxon Rank Sum Test – pairwise

Groups	P–value	Adj. p–value
1–2	0.0000	0.0000
2–3	0.0258	0.0387

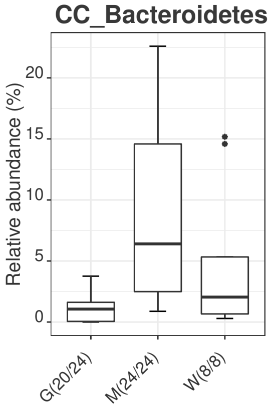

Wilcoxon Rank Sum Test – pairwise

Groups	P–value	Adj. p–value
1–2	0.0000	0.0000
2–3	0.0368	0.0552
