# Supplementary material for: The yellow perch (Perca flavescens) microbiome revealed resistance to colonisation mostly associated with neutralism driven by rare taxa under cadmium disturbance
Source: Anim Microbiome. 2021 Jan 5;3:3. doi: 10.1186/s42523-020-00063-3 (PMC7934398; doi:10.1186/s42523-020-00063-3)
Supplement: Supplementary file 12 — Additional file 12: Figure S6. Water and host-microbial interactions network over time and between communities. This figure summarises the dynamic of interactions of water with the host microbiome networks. Each node size in the network is proportional to the average of the OTUs relative abundance in all samples. These networks are based on significant Spearman coefficients and were constructed using R scripts and Cytoscape software. [file 42523_2020_63_MOESM12_ESM.pdf]

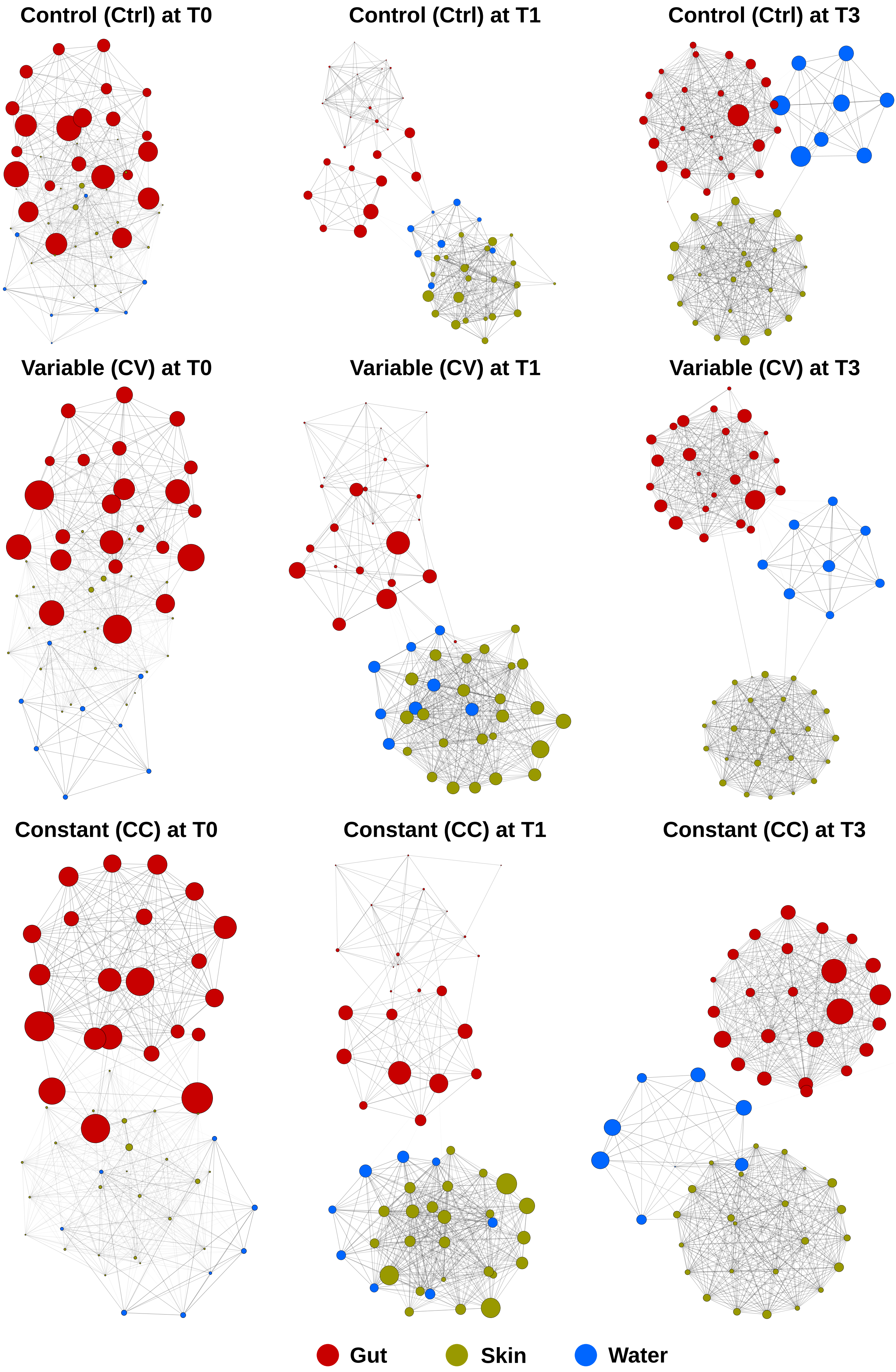
Control (Ctrl) at T0
Control (Ctrl) at T1
Control (Ctrl) at T3
Variable (CV) at T0
Variable (CV) at T1
Variable (CV) at T3
Constant (CC) at T0
Constant (CC) at T1
Constant (CC) at T3
Gut
Skin
Water
