# Supplementary material for: The yellow perch (Perca flavescens) microbiome revealed resistance to colonisation mostly associated with neutralism driven by rare taxa under cadmium disturbance
Source: Anim Microbiome. 2021 Jan 5;3:3. doi: 10.1186/s42523-020-00063-3 (PMC7934398; doi:10.1186/s42523-020-00063-3)
Supplement: Supplementary file 13 — Additional file 13: Figure S7. Statistical analysis of the closeness centrality in the water and host-microbiome networks. The comparison of centrality assessed with nodes closeness centrality and represented with violin plots indicate that the average of connections is significantly higher in the skin compared to the water and gut microbiomes in all treatments at T0 before disturbance. However, at T1 the centrality of node converged (which means not significantly different) between the water and skin microbiome for cadmium-treated groups. At T3, the centrality of node converged between the gut and skin microbiome in the control group. The average of nodes’ degree computed with Network Analyzer was compared using the Kruskal-Wallis test followed by Benjamini-Hochberg test. The value of 0.05 is the threshold of B-H p-value significance. [file 42523_2020_63_MOESM13_ESM.pdf]

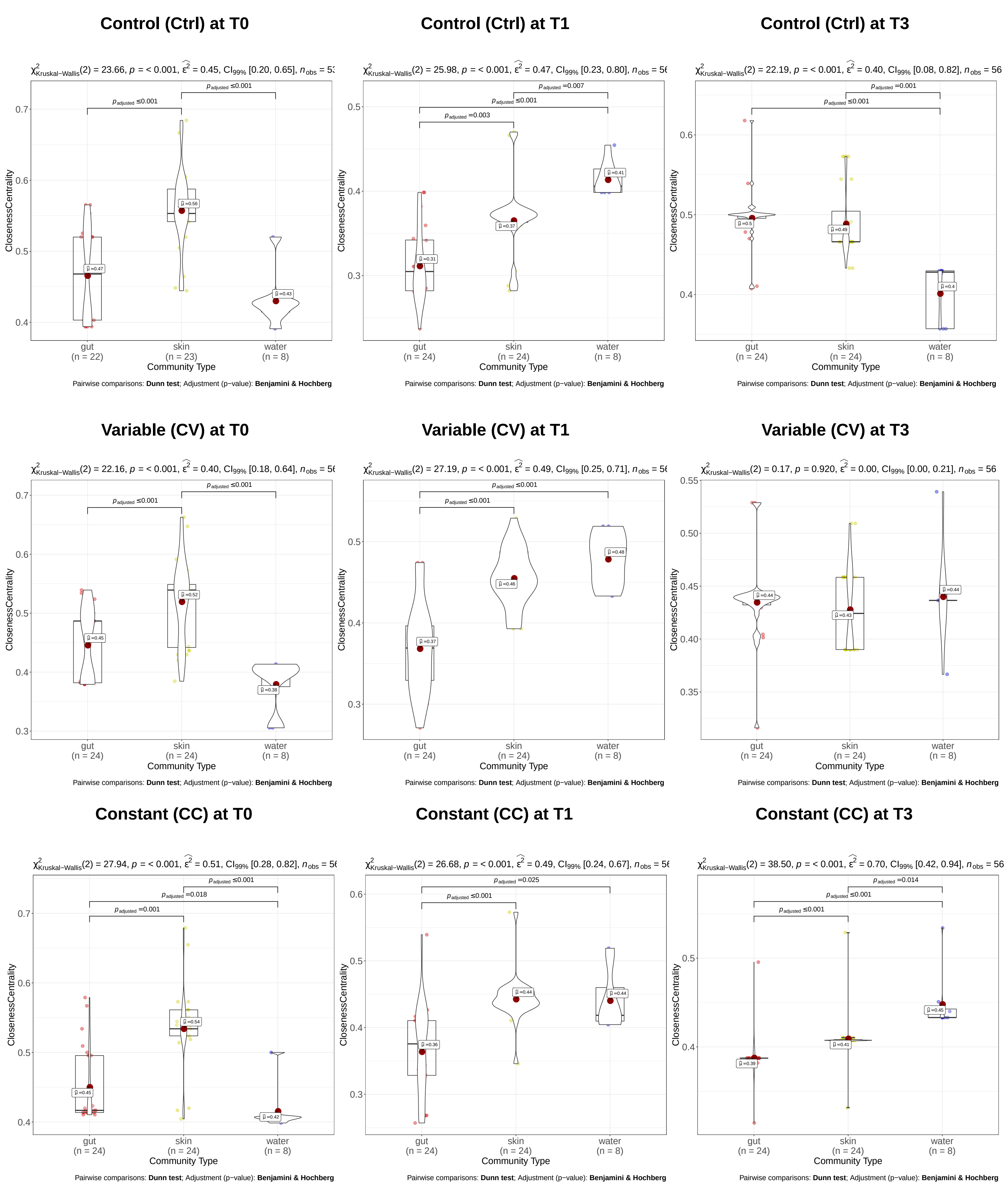
Control (Ctrl) at T0
χ²Kruskal−Wallis(2) = 23.66, p = < 0.001, ε̂² = 0.45, CI99% [0.20, 0.65], nobs = 53
padjusted ≤0.001
padjusted ≤0.001
μ̂ =0.47
μ̂ =0.56
μ̂ =0.43
ClosenessCentrality
gut (n = 22)
skin (n = 23)
water (n = 8)
Community Type
Pairwise comparisons: Dunn test; Adjustment (p−value): Benjamini & Hochberg
Control (Ctrl) at T1
χ²Kruskal−Wallis(2) = 25.98, p = < 0.001, ε̂² = 0.47, CI99% [0.23, 0.80], nobs = 56
padjusted =0.007
padjusted ≤0.001
padjusted =0.003
μ̂ =0.31
μ̂ =0.37
μ̂ =0.41
ClosenessCentrality
gut (n = 24)
skin (n = 24)
water (n = 8)
Community Type
Pairwise comparisons: Dunn test; Adjustment (p−value): Benjamini & Hochberg
Control (Ctrl) at T3
χ²Kruskal−Wallis(2) = 22.19, p = < 0.001, ε̂² = 0.40, CI99% [0.08, 0.82], nobs = 56
padjusted =0.001
padjusted ≤0.001
μ̂ =0.5
μ̂ =0.49
μ̂ =0.4
ClosenessCentrality
gut (n = 24)
skin (n = 24)
water (n = 8)
Community Type
Pairwise comparisons: Dunn test; Adjustment (p−value): Benjamini & Hochberg
Variable (CV) at T0
χ²Kruskal−Wallis(2) = 22.16, p = < 0.001, ε̂² = 0.40, CI99% [0.18, 0.64], nobs = 56
padjusted ≤0.001
padjusted ≤0.001
μ̂ =0.45
μ̂ =0.52
μ̂ =0.38
ClosenessCentrality
gut (n = 24)
skin (n = 24)
water (n = 8)
Community Type
Pairwise comparisons: Dunn test; Adjustment (p−value): Benjamini & Hochberg
Variable (CV) at T1
χ²Kruskal−Wallis(2) = 27.19, p = < 0.001, ε̂² = 0.49, CI99% [0.25, 0.71], nobs = 56
padjusted ≤0.001
padjusted ≤0.001
μ̂ =0.37
μ̂ =0.46
μ̂ =0.48
ClosenessCentrality
gut (n = 24)
skin (n = 24)
water (n = 8)
Community Type
Pairwise comparisons: Dunn test; Adjustment (p−value): Benjamini & Hochberg
Variable (CV) at T3
χ²Kruskal−Wallis(2) = 0.17, p = 0.920, ε̂² = 0.00, CI99% [0.00, 0.21], nobs = 56
μ̂ =0.44
μ̂ =0.43
μ̂ =0.44
ClosenessCentrality
gut (n = 24)
skin (n = 24)
water (n = 8)
Community Type
Pairwise comparisons: Dunn test; Adjustment (p−value): Benjamini & Hochberg
Constant (CC) at T0
χ²Kruskal−Wallis(2) = 27.94, p = < 0.001, ε̂² = 0.51, CI99% [0.28, 0.82], nobs = 56
padjusted ≤0.001
padjusted =0.018
padjusted =0.001
μ̂ =0.45
μ̂ =0.54
μ̂ =0.42
ClosenessCentrality
gut (n = 24)
skin (n = 24)
water (n = 8)
Community Type
Pairwise comparisons: Dunn test; Adjustment (p−value): Benjamini & Hochberg
Constant (CC) at T1
χ²Kruskal−Wallis(2) = 26.68, p = < 0.001, ε̂² = 0.49, CI99% [0.24, 0.67], nobs = 56
padjusted =0.025
padjusted ≤0.001
μ̂ =0.36
μ̂ =0.44
μ̂ =0.44
ClosenessCentrality
gut (n = 24)
skin (n = 24)
water (n = 8)
Community Type
Pairwise comparisons: Dunn test; Adjustment (p−value): Benjamini & Hochberg
Constant (CC) at T3
χ²Kruskal−Wallis(2) = 38.50, p = < 0.001, ε̂² = 0.70, CI99% [0.42, 0.94], nobs = 56
padjusted =0.014
padjusted ≤0.001
padjusted ≤0.001
μ̂ =0.39
μ̂ =0.41
μ̂ =0.45
ClosenessCentrality
gut (n = 24)
skin (n = 24)
water (n = 8)
Community Type
Pairwise comparisons: Dunn test; Adjustment (p−value): Benjamini & Hochberg
