# Supplementary material for: The yellow perch (Perca flavescens) microbiome revealed resistance to colonisation mostly associated with neutralism driven by rare taxa under cadmium disturbance
Source: Anim Microbiome. 2021 Jan 5;3:3. doi: 10.1186/s42523-020-00063-3 (PMC7934398; doi:10.1186/s42523-020-00063-3)
Supplement: Supplementary file 14 — Additional file 14: Figure S8. Skin microbiome networks built with SPIEC method. The SPIEC-EASI (SParse Inverse Covariance Estimation for Ecological Association Inference) method [55] implemented in R was applied using Meinshausen-Buhlmann’s neighbourhood selection (MB) method to estimate the inverse covariance matrix. The OTUs having low frequency occurrence (occurrence <=3) were dummied in one synthetic OTUs (black node). Red and grey edges represent negative and positive regression coefficients of the inverse covariance matrix. The node size in the network is proportional to the average of an OTU relative abundance in all samples. These networks were visualized using Cytoscape software. [file 42523_2020_63_MOESM14_ESM.pdf]

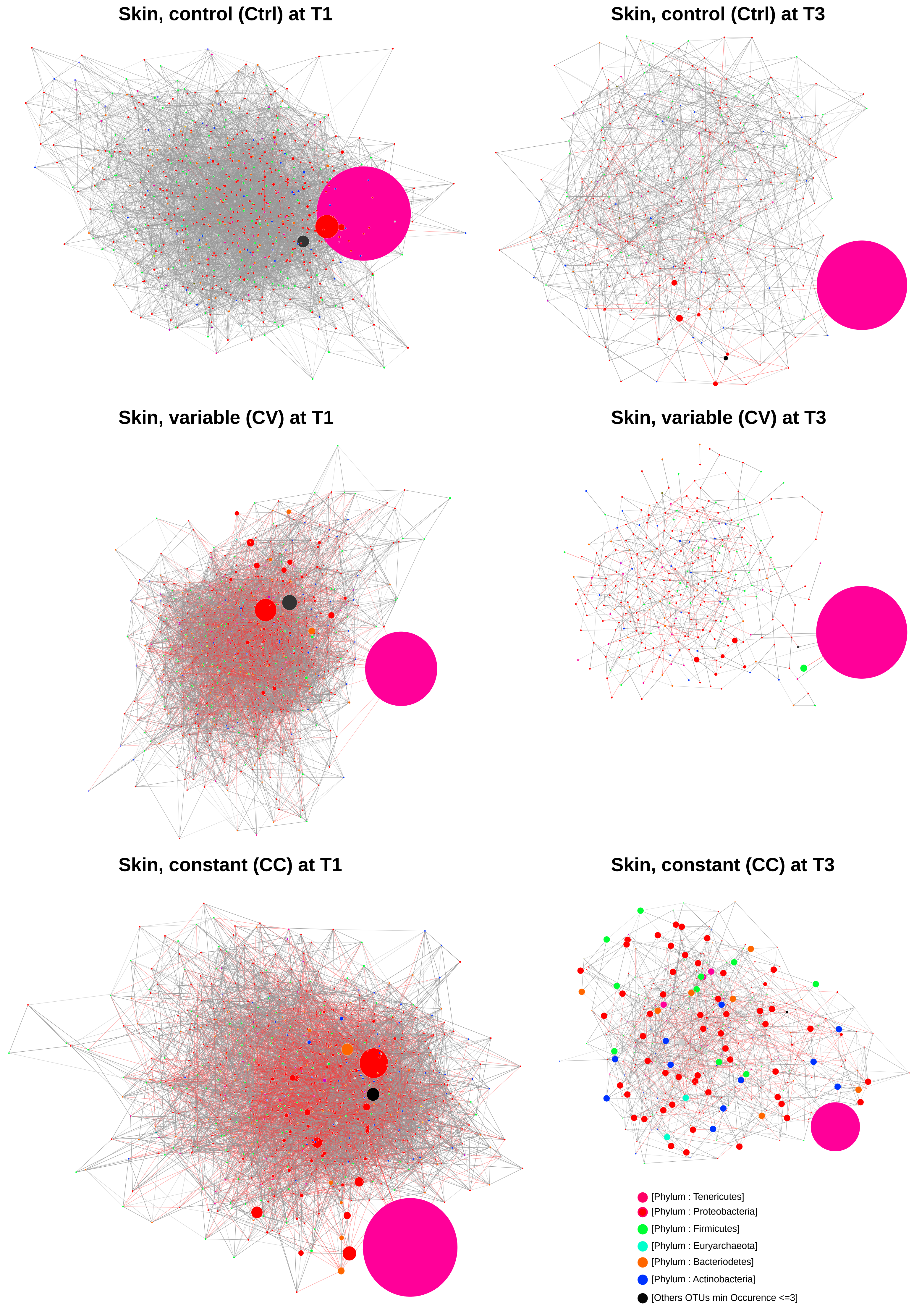
Skin, control (Ctrl) at T1
Skin, control (Ctrl) at T3
Skin, variable (CV) at T1
Skin, variable (CV) at T3
Skin, constant (CC) at T1
Skin, constant (CC) at T3
[Phylum : Tenericutes]
[Phylum : Proteobacteria]
[Phylum : Firmicutes]
[Phylum : Euryarchaeota]
[Phylum : Bacteriodetes]
[Phylum : Actinobacteria]
[Others OTUs min Occurence <=3]
