# Supplementary material for: The yellow perch (Perca flavescens) microbiome revealed resistance to colonisation mostly associated with neutralism driven by rare taxa under cadmium disturbance
Source: Anim Microbiome. 2021 Jan 5;3:3. doi: 10.1186/s42523-020-00063-3 (PMC7934398; doi:10.1186/s42523-020-00063-3)
Supplement: Supplementary file 15 — Additional file 15: Figure S9. Distribution of neutrality versus abundance cut-off and goodness of fit. This figure shows the variation of neutral OTUs percentage (Y-axis) and goodness of fit predicted by NLS models using 12 cut-offs thresholds of relative abundance percentages (gg facet panels) in the entire metacommunity at T1 and T3. [file 42523_2020_63_MOESM15_ESM.pdf]

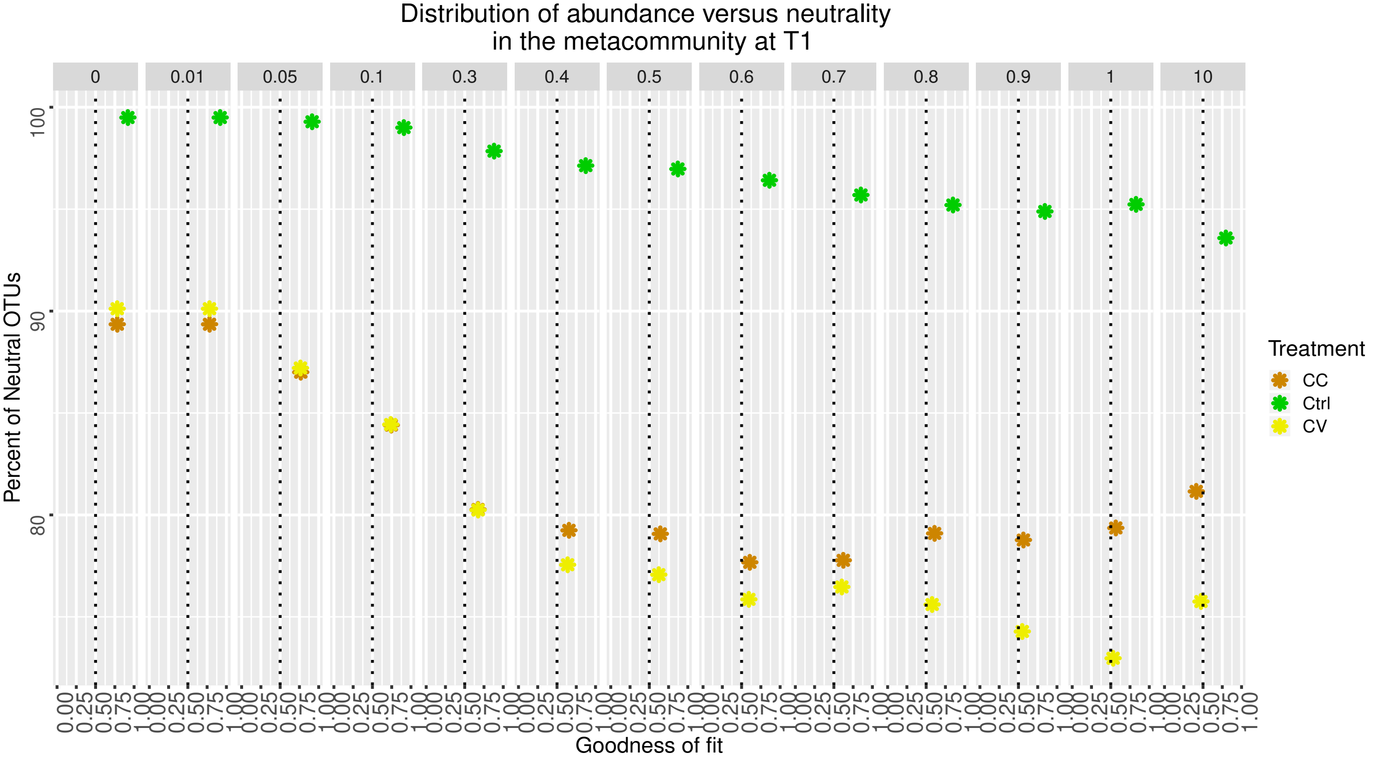
Distribution of abundance versus neutrality
in the metacommunity at T1
0
0.01
0.05
0.1
0.3
0.4
0.5
0.6
0.7
0.8
0.9
1
10
Percent of Neutral OTUs
100
90
80
0.00 0.25 0.50 0.75 1.00
Goodness of fit
Treatment
CC
Ctrl
CV

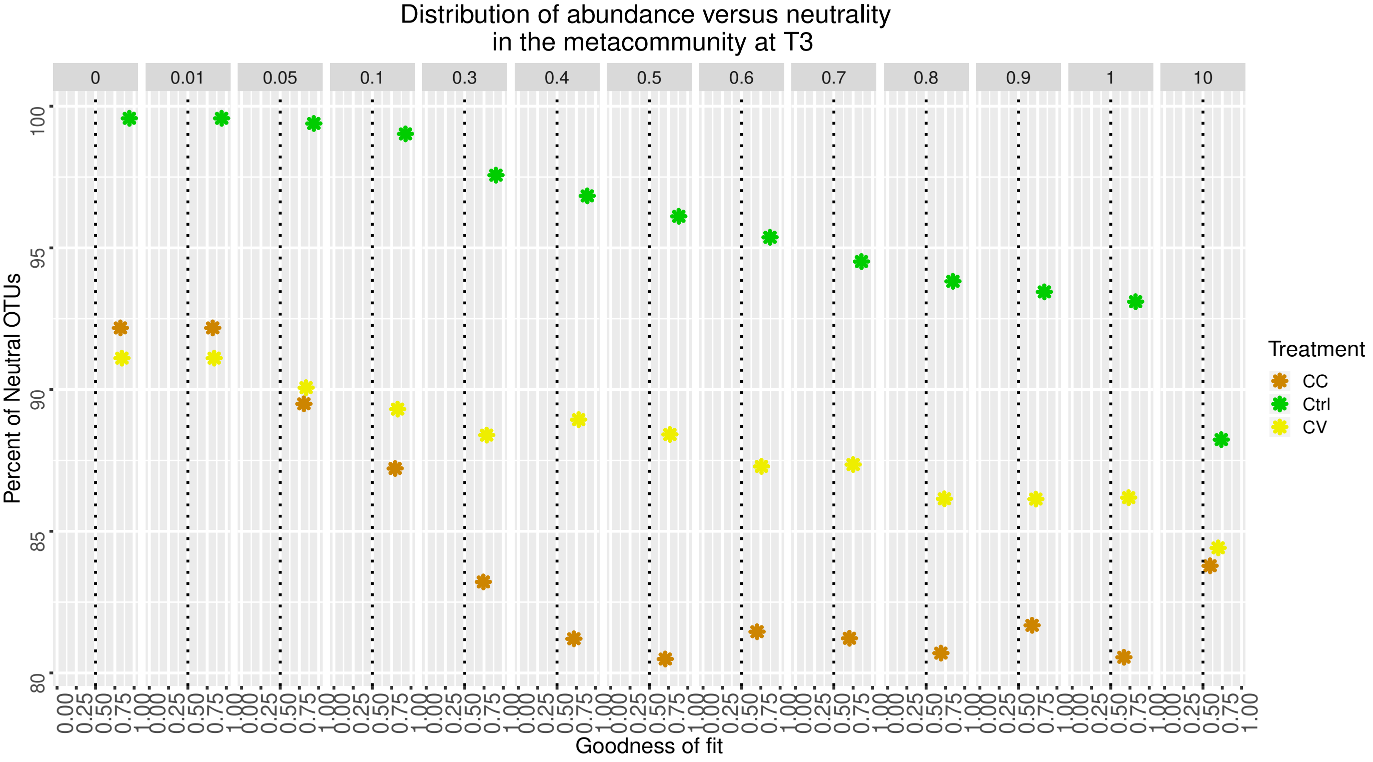
Distribution of abundance versus neutrality
in the metacommunity at T3
0
0.01
0.05
0.1
0.3
0.4
0.5
0.6
0.7
0.8
0.9
1
10
Percent of Neutral OTUs
100
95
90
85
80
0.00 0.25 0.50 0.75 1.00
Goodness of fit
Treatment
CC
Ctrl
CV
