# Supplementary material for: The yellow perch (Perca flavescens) microbiome revealed resistance to colonisation mostly associated with neutralism driven by rare taxa under cadmium disturbance
Source: Anim Microbiome. 2021 Jan 5;3:3. doi: 10.1186/s42523-020-00063-3 (PMC7934398; doi:10.1186/s42523-020-00063-3)
Supplement: Supplementary file 16 — Additional file 16: Supplementary file 1. [file 42523_2020_63_MOESM16_ESM.docx]

**Supplementary file 1**

**Materials and Methods (supplementary information)**

**Metal concentration in water and fish liver.**

Metal traces concentrations (Cd, Cu and Zn) in water and liver were determined with the ICPMS (Ionization Coupled Mass spectrometry) technology at Chemical department of Laval University for T0 and T1, then at INRS (Institut National de la Recherche Scientifique) for T1-T3. Before ICPMS analysis, the Cd in water samples was fixed by adding 4% of nitric acid. This analysis was performed every week until the end of Cd exposure regimes. Liver samples stored in Eppendorf^®^ tubes were weighed before and after lyophilisation. Freeze-dried samples were digested using purified nitric acid and kept at room temperature upon five days. The digestion protocol was adapted from Borgmann [1] and recently modified [2]. The method validity was always verified at INRS using internal standards (DOLT-4, Dogfish liver) certified reference material for trace metals, National Research Council of Canada (NRC-CNRC); TORT-3, lobster hepatopancreas (NRC-CNRC, Ottawa, ON). The digested samples were diluted with distilled water and the concentration of Cd, Cu and Zn were measured using ICPMS machine at INRS. The metals concentrations were analysed using two-way analysis of variance (ANOVA) of two independent factors, time and treatment. Depending on the normality of the metal’s concentration distribution inspected with the Shapiro test, the interactions between time and treatment factors were analysed using the Tukey’s test and Wilcoxon rank test

**Fish rearing**.

Two weeks after hatching, Yellow perch juveniles (size/weight) were transported in controlled temperature from Kinmount Fish Farm (Ontario, Canada) to the LARSA (Laboratoire de Recherche en Sciences Aquatiques). The perch were kept in four 1 m^3^ indoor tanks for quarantine and acclimation in open circuit. They were held under natural photoperiod conditions, and the temperature was set at 20°C. After three weeks of acclimation, the temperature was gradually lowered 1°C every 48 hours until it reached 11°C. The perch were randomly distributed in 24 tanks (50 fish per tank) of 36 L each tank was an independent filtering system circuit. The fish juveniles were daily feed from the beginning to the end of the experience. After an acclimation period of two weeks in independent tanks, selection regimes of Cadmium started.

**Ethics statement.**

This experiment and protocol were approved by the Ethics Committee for the Use of Animals of Laval University (number 027/2015). All methods were carried out by the approved guidelines.

**Exposure regimes to cadmium**.

Control and Cd-treated tanks were randomly labelled. The experiment was designed for two cadmium exposure regimes (8 tanks per regime), and one negative control regime (8 tanks). In treated tanks, fish were exposed to cadmium chloride (CdCl_2_) provided by Sigma-Aldrich (> 99.9% purity). The Cadmium was dissolved in water following an increasing concentration gradient. For the constant concentration regime (CC), initially added at 0.8 ppb, Cd concentration was gradually increased every five days to reach a maximal concentration at the end of the first month (T1). This maximal concentration was maintained two months until the end of treatment (third month, T3). For the variable concentration regime (CV), initially added at 0.6 ppb, Cd concentration was increased every five days to reach the maximal concentration at the end of treatment (third month, T3). The maximal Cd concentration was empirically set at nine ug/L as it is the highest Cd concentration tolerated by yellow perch in contaminated Canadian lakes [3]. The Cd exposure was stopped after three months at T3. The resilience of fish and water microbiota was the subject of the next study, so the experiment was extended two months after T3. Through all the experiment period, to maintain viable conditions for Perch in each water tank, faecal and food particles were daily cleaned using specific pressing tubes of each experimental conditions. Then, a volume of 15 L of water was renewed two times a week in each tank, according to the Cd concentration of each experimental group. Water pH was adjusted five days per week, ammonia (NH_3_) and nitrogen dioxide (NO_2_) were periodically stabilised.

**Mucosa sampling of perch skin and gut**.

After a long exposure period, fish were anaesthetised using MS-222 (2 mg/L) by the animal wellbeing guidelines for fish anaesthesia. Nine fishes were sampled per tank, thus representing three biological replicates per tank, each replicate is a pool of three fishes. In a sterile platform, underneath a laminar flow hood, with sterile cotton swabs, the skin mucus (from jaws to caudal-fin lobe) was swabbed, three swabs per replicate were stored in 2mL sterile microcentrifuge tube and directly stored at -80 C. Length to the fork and weight of each fish were measured after skin mucus sampling. Once dissected, intestine and liver of each three fish were pooled in 2mL sterile microcentrifuge tubes and stored at -80 C. For intestine samples, to extract RNA of a subsequent transcriptomic project, 1 mL of Trizol (SIGMA-ALDRICH TRI Reagent) was added to each sample before the cryogenic storage. A total of 432 host-microbiota samples were collected for this study, 216 (3 times x 3 regimes x 8 tanks x 3 replicates) of swabbed skin mucus and 216 (3 times x 3 regimes x 8 tanks x 3 replicates) of gut tracts samples.

**Water tanks sampling**

Sampled water was stored in the sterile bottle (Nalgene), 2 litres per tank. The water filtration protocol was performed using a polycarbonate membrane (0.22 μm) (). For each sampling time (T0, T1, and T3), we collected eight tanks per regime, two replicates per tank, and two filters (0.22 μm) per replicate. In total, 144 filters (3 times x 3 regimes x 8 tanks x 2 replicates) were conserved from this study.

**DNA extraction**

The Extraction was performed at the Institut de Biologie Intégrative et des Systèmes (IBIS), Université Laval (Québec, QC). DNA was extracted from all skin mucus and water using the Qiagen DNeasy Blood and Tissue kit according to the manufacturer's instructions with slight adjustment by adding 40 µL lysozyme for proteins digestion and eight µL of RNase for RNA degradation. Negative DNA extraction was performed on a sterile swab and ionised water to discard any potential bias of contamination; any significant concentration of DNA was quantified from white extraction. For all intestine samples, after RNA extraction for a transcriptomic project, the DNA was extracted from TRIzol organic phase using BEB (back extraction buffer) and PCI (phenol/chloroform/isoamyl alcohol 25:24:1) solution (protocol available under request).

**Universal gene Amplification**.

The DNA was amplified using universal primers specific to the V3-V4 hypervariable region of the rRNA 16S gene. Amplification was primed with 347_f 5′-ACACTCTTTCCCTACACGACGCTCTTCCGATCTGGAGGCAGCAGTRRGGAAT-3′, and 805_r 5′-GTGACTGGAGTTCAGACGTGTGCTCTTCCGATCTCTACCRGGGTATCTAATCC-3′ primers using Q5 High-Fidelity DNA polymerase (NEB). The PCR was carried out in a total volume of 50 µL that contains 10 µL of 5X buffer (NEB Q5 Kit), 10 µL of 5X GC enhancer (NEB Q5 Kit), 0.25 µM of each primer, 1 µL of dNTPs (200 µM), 25.5 µL of sterile ultra-pure water, 1 µL of Q5 High-Fidelity DNA polymerase (NEB) and 2 µL of template DNA. The PCR started with an initial denaturation at 98°C for 30 s followed by ten cycles of denaturation at 98°C for 10 s, annealing at 60°C for 10s, extension at 72°C for the 30s. An additional 25 cycles were as follows denaturation at 98°C for 10 s, annealing at 64°C for 10 s, extension at 72°C for the 30s followed by a final extension step at 72°C for 2 min. Amplified DNA was purified with beads (Axygen) to eliminate primers, dimers and phenols (protocol available under request). DNA concentration and quality were verified by electrophoresis on 2% agarose gel and by Nanodrop 1000 (Thermo Fisher Scientific).

**Amplicons Libraries**

The purified cDNA product was used as a template for the library preparation. The second PCR was carried out in a total volume of 50 µL that contains 10 µL of 5X buffer (NEB Q5 Kit), 10 µL of 5X GC enhancer (NEB Q5 Kit), 3 µL of the forward library (1 µM), 3 µL of reverse library index (1 µM), 1 µL of dNTPs (200 µM), 20 µL of sterile ultra-pure water, 1 µL of Q5 High-Fidelity DNA polymerase (NEB) and 1 µL of template DNA. A combination of 768 indexes was conceived for 768 samples. The PCR started with an initial denaturation at 98°C for 30 s followed by ten cycles of denaturation at 98°C for 10 s, annealing at 64°C for 10s, extension at 72°C for the 30s. An additional 25 cycles were as follows denaturation at 98°C for 10 s, annealing at 64°C for 10 s, extension at 72°C for the 30s followed by a final extension step at 72°C for 2 min. Amplified DNA was purified with beads (Axygen). Final amplified DNA was verified by electrophoresis on 2% agarose gel, and double-strand DNA concentration was quantified by fluorescence using Quant-iT™ PicoGreen™ dsDNA Assay Kit (Thermo Fischer Scientific).

**References**

1. Borgmann U, Néron R, Norwood WP. Quantification of bioavailable nickel in sediments and toxic thresholds to Hyalella azteca. *Environ Pollut* 2001; **111**: 189–198.

2. Pierron F, Bourret V, St-Cyr J, Campbell PGC, Bernatchez L, Couture P. Transcriptional responses to environmental metal exposure in wild yellow perch (Perca flavescens) collected in lakes with differing environmental metal concentrations (Cd, Cu, Ni). *Ecotoxicology* 2009; **18**: 620–631.

3. Couture P, Rajotte JW, Pyle G. Seasonal and regional variations in metal contamination and condition indicators in yellow perch (Perca flavescens) along two polymetallic gradients. III. Energetic and physiological indicators. 2008.
