# Supplementary material for: The yellow perch (Perca flavescens) microbiome revealed resistance to colonisation mostly associated with neutralism driven by rare taxa under cadmium disturbance
Source: Anim Microbiome. 2021 Jan 5;3:3. doi: 10.1186/s42523-020-00063-3 (PMC7934398; doi:10.1186/s42523-020-00063-3)
Supplement: Supplementary file 17 — Additional file 17: Supplementary file 2. [file 42523_2020_63_MOESM17_ESM.docx]

**Supplementary File 2. A brief description of the sample groups of host microbiota and water**

| **Experimental group** | **Habitat type** | **Sampling time** | **Sample number** | **Replicate description** | **Sample Description** | **Sampling method** | **DNA extraction protocol** |
| --- | --- | --- | --- | --- | --- | --- | --- |
| WT.Ctrl | Water | T0, T1, T3, | 1x8x8 | A pool of two membrane filters | Control | Peristaltic filtration | DNA easy Blood and tissue |
| WT.CC | Water | T0, T1, T3 | 1x8x8 |  | Cadmium constant concentration | Peristaltic filtration | DNA easy Blood and tissue |
| WT.CV | Water | T0, T1, T3 | 1x8x8 |  | Cadmium variable concentration | Peristaltic filtration | DNA easy Blood and tissue |
| MT.Ctrl | Skin mucus | T0, T1, T3 | 3x8x4 | A pool of three cotton swabs from three fish | Control | Sterile cotton swabs | DNA easy Blood and tissue |
| MT.CC | Skin mucus | T0, T1, T3 | 3x8x4 |  | Cadmium constant concentration | Sterile cotton swabs | DNA easy Blood and tissue |
| MT.CV | Skin mucus | T0, T1, T3 | 3x8x4 |  | Cadmium variable concentration | Sterile cotton swabs | DNA easy Blood and tissue |
| GT.Ctrl | Gut mucus | T0, T1, T3 | 3x8x4 | A pool of three intestines from three different individual fish | Control | Sterile dissection | BEB extraction |
| GT.CC | Gut mucus | T0, T1, T3 | 3x8x4 |  | Cadmium constant concentration | Sterile dissection | BEB extraction |
| GT.CV | Gut mucus | T0, T1, T3 | 3x8x4 |  | Cadmium variable concentration | Sterile dissection | BEB extraction |
