# Supplementary material for: The yellow perch (Perca flavescens) microbiome revealed resistance to colonisation mostly associated with neutralism driven by rare taxa under cadmium disturbance
Source: Anim Microbiome. 2021 Jan 5;3:3. doi: 10.1186/s42523-020-00063-3 (PMC7934398; doi:10.1186/s42523-020-00063-3)
Supplement: Supplementary file 18 — Additional file 18: Supplementary file 3. [file 42523_2020_63_MOESM18_ESM.docx]

**DNA extraction from TRIZOL from the organic phase after RNA extraction**

1. After having taken the aqueous phase with RNA, spin down the tubes which contain the interphase/organic phase with TRIzol at 12,000 x g for 5 min. At 4 C. Transfer the organic phase in a new tube, do not take any aqueous phase, which would contaminate the DNA sample with RNA. Now the interphase contains the DNA. At this point, we can store your samples at 4 C or -80 C for days to weeks.
2. Add 0.025-0.5 ml of the BEB (back extraction buffer) per 1 ml of TRIzol used for the RNA extraction to each tube. 1 ml of TRIzol was used for our samples. Mix intensively at least 3 min. by inversion or put on a shaker for 10 min, at room temperature, 600 rpm
3. Spin tubes at 12,000 x g for 30 min at room temperature.
4. Transfer upper aqueous phase if we see one. We will probably not.
5. Add 0.4 ml isopropanol per 1 ml TRIzol used for initial RNA isolation. Mix and incubate for 5 min at RT.
6. Then we Spin samples at 12,000 x g for 15 min at 4 C.
7. Remove supernatant, keep pellet with DNA. Add 0.5 ml of cold ethanol 70% per 1 ml TRIzol used for RNA extraction and wash pellet by inversion.
8. Spin down at 12,000 g for 15 min. at 4 C. Remove ethanol and let air dry at 38 ^o^C overnight. Dissolve pellet in about 400 ul of 1x TE buffer. DNA can be stored at 4 C. This DNA will perform fine in many reactions.

**Washing steps with phenol, chloroform and isoamylalcohol**

1. Add an equal volume of PCI (400ul) directly to the tube.
2. Mix the organic and aqueous phases intensively by inverting and shake 10 min, at room temperature, 600 rpm
3. Centrifuge at 10,000-12,000 x g for 15 min. at RT to separate the phases. The PCI will be located at the bottom of the tube, while the dissolved DNA will remain above the gel.
4. Take new tubes, transfer aqueous phase to a new tube.
5. Repeat Step 9-12 once.
6. Add the same volume (about 200 ul) of *chloroform-isoamylalcohol* (or chloroform only) to the upper aqueous phase and centrifuge as in step 11.
7. Transfer the upper aqueous phase to a new 1.5 – 2.0 ml microfuge tube.
8. Add 20 μl (10% of the volume) of 3M Sodium Acetate pH 5.2–7.0. Mix and add 2 to 2.5 volumes of cold 95-100% of ethanol (96%). Mix, and might see the DNA coming out of the solution.
9. Spin down the DNA for 10-15 minutes in a micro-centrifuge at full speed.
10. Discard the liquid and add 100 μl of cold 70% ethanol. Spin down DNA for 10-15 minutes in a micro-centrifuge at full speed.

19. Discard the liquid and air dry at 38 ^o^C overnight.

Resuspend pellet in 1 x TE or DNAse free water, let DNA stay at RT for 5-6 hours or overnight

20. Then measure OD and run 1% agarose gel to check DNA’s quality. Then store DNA at 4

C until use.

**NB. Never vortex samples but mix by inversion (genomic DNA) (unless stated otherwise). Store genomic DNA at 4 C (not at –20 C).**

**To be prepared**:

*Back extraction buffer (BEB)*

For 250 ml BEB (take precautions – toxic substances -):

Take 150 ml of millipore water and dissolve

- 118.2 g of Guanidine Thiocyanate (FW 118.2) = 4 M
- 3.68 g of Sodium Citrate NaCi (FW 294.1) = 50 mM
- 30.29 g of Tris (free base) Tris (FW 121.14) = 1M
- add additional millipore water until the volume is 250 ml
- sterilise bottle

# 3 M Sodium acetate pH 5.2 –7

*1 x TE buffer pH 8.0*

*phenol/chloroform/isoamylalcohol 25:24:1 (PCI) ( very toxic)*
